# A Life-Cycle Model of Human Social Groups Produces a U-Shaped Distribution in Group Size

**DOI:** 10.1371/journal.pone.0138496

**Published:** 2015-09-18

**Authors:** Gul Deniz Salali, Harvey Whitehouse, Michael E. Hochberg

**Affiliations:** 1 Institut des Sciences de l'Evolution, Université de Montpellier, Place E Bataillon, 34095, Montpellier, Cedex 5, France; 2 Department of Anthropology, University College London, 14 Taviton Street, London, WC1H 0BW, United Kingdom; 3 Institute of Cognitive and Evolutionary Anthropology, University of Oxford, Oxford, OX2 6PN, United Kingdom; 4 Santa Fe Institute, 1399 Hyde Park Road, Santa Fe, New Mexico, 87501, United States of America; Tianjin University of Technology, CHINA

## Abstract

One of the central puzzles in the study of sociocultural evolution is how and why transitions from small-scale human groups to large-scale, hierarchically more complex ones occurred. Here we develop a spatially explicit agent-based model as a first step towards understanding the ecological dynamics of small and large-scale human groups. By analogy with the interactions between single-celled and multicellular organisms, we build a theory of group lifecycles as an emergent property of single cell demographic and expansion behaviours. We find that once the transition from small-scale to large-scale groups occurs, a few large-scale groups continue expanding while small-scale groups gradually become scarcer, and large-scale groups become larger in size and fewer in number over time. Demographic and expansion behaviours of groups are largely influenced by the distribution and availability of resources. Our results conform to a pattern of human political change in which religions and nation states come to be represented by a few large units and many smaller ones. Future enhancements of the model should include decision-making rules and probabilities of fragmentation for large-scale societies. We suggest that the synthesis of population ecology and social evolution will generate increasingly plausible models of human group dynamics.

## Introduction

Human societies exhibit different social and cultural organizations and technologies [1]. Scholars have argued that human societies have evolved by augmenting genetic adaptation with sociocultural differentiation and technology [1,2]. Human political, social or religious groups furnish examples of adaptations to different niches by cultural differentiation. They have heritable characteristics and are subject to selection, such that those social and/or cultural features providing greater competitive fitness to their groups tend to prevail [3,4]. In this respect, sociocultural groups resemble living organisms: they emerge, grow, interact, reproduce, and eventually dissolve or die. In this study, we develop a lifecycle theory for the emergence and evolution of human sociocultural groups and investigate the factors affecting patterns in group dominance and coexistence.

Human societies exhibit uniquely rich cultural diversity, with some 6909 extant languages and over 3814 distinct cultures worldwide [5]. These cultural groups also differ in their political complexity. With respect to ethnographic and archaeological data, human political organization has been classified into four distinctive forms in terms of characteristics and complexity [6–8]. According to this classification, for most of their existence modern humans lived in small bands of groups in villages that were politically autonomous. However, roughly 10,000 years ago quantitative changes (larger population sizes/densities) gave rise to social change when villages began aggregating into larger and more complex polities [9,10]. Tribes emerged from small bands [8], characterized as usually egalitarian societies where leadership was ephemeral and decisions were made by periodic gatherings of social units in communal feasts and festivals [8]. With increasing demographic complexity, tribes evolved into chiefdoms where the local groups were centralized under inherited leadership [8]. Decision-making was a faster process compared to decentralized societies [11]. Finally, growing chiefdoms evolved into states, i.e. centralized political structures consisting of specialized administrative organizations [12,13]. The hierarchical nesting of social units in a complex society incorporated local leaders as part of centralized systems of regulation and control [14,15].

Although the progressive trend towards larger and more complex societies has been documented all around the world, the pattern and tempo of political evolution varied with numerous examples of societies that have decreased in complexity or eventually collapsed leading to a cycling process of rise and fall of complex societies throughout history [16–18]. The actual mode and tempo of political evolution are controversial topics regarding whether states emerged gradually from quantitative changes in chiefdom societies—“gradualism”—or if their appearance was the result of abrupt and qualitative change—analogous to a “punctuated equilibrium” in population biology [19]. Recently, studies have shown that in South-East Asia and the Pacific, political complexity rises and falls in a sequence of small steps, and while decreases from more to less hierarchical societies is possible, increases have been more common [6,7].

Transitions among different levels of socio-political complexity can be affected by environmental, demographic and cultural factors, and their interactions [5,20]. For instance, it has been argued that the emergence of complex societies was influenced by climatic conditions, specifically that harsh climate during the Pleistocene prevented the appearance of agriculture [21]. According to this view, while the Pleistocene was characterized by dispersed resources leading to subsistence strategies based on hunting and gathering in small bands, the ameliorated conditions of climate in the Holocene was amenable to humans domesticating crops and animals, which in turn resulted in aggregation and increasing population densities [14]. Moreover, geographic features played a key role by either allowing or obstructing information flow between populations. Specifically, physical barriers caused by topographic heterogeneity is expected to lead to little or no information flow between groups and cultural group divergence [20]. When geography permits the flow of information between sub-populations, cultural evolution can proceed at higher rates, potentially resulting in the establishment of more complex societies. For example, in Eurasia the size and orientation of land allowed human cultures to spread by diffusion, resulting in the early development of agriculture and complex societies [12].

There is some evidence that the shift from small-scale to large-scale societies that accompanied the emergence of agriculture also required a change in the kinds of rituals necessary for building cultural groups. Ancestral foraging bands were bound together by relational ties between individuals who knew each other personally. State formation was a process through which people came to be bonded with much larger collectives of with an impersonal membership. In these much larger groups, members were bound together by categorical rather than relational ties. It has been argued that these two contrasting forms of group alignment correspond to a fundamental divergence in modes of religious experience and practice (hereafter referred to as “modes theory”), whereby religious organizations tend to cluster around two socio-political “attractor positions” or “modes”: one localized, non-centralized but highly cohesive (“imagistic”) and the other large-scale, diffuse, and hierarchical [22–24]. Imagistic groups engage in highly arousing and infrequent rituals (such as the traumatic initiations of various cults, experiences of collective possession and altered states of consciousness) where individuals gather episodic memories and reinforce in-group cohesion. In contrast, individuals in a doctrinal group undertake highly routinized ritual action, collecting conceptually complex religious teachings taught by religious leaders and standardized through public repetition. The codification of doctrine verbally and in text allows faster spread of religious beliefs and practices, leading to large-scale and centralized religious communities [24].

Although the modes theory was originally intended to explain observed patterns in religious groups, it has been shown to apply to a much wider range of groups with collective rituals, ranging from military units to sports clubs and university fraternities [25,26]. As such, the modes theory can be used as a general framework to investigate the dynamics of different human social and political organizations. Recent archaeological evidence from the early Neolithic site at Çatalhöyük suggests that the emergence of the doctrinal mode may have been linked to the dawn of agriculture and the subsequent emergence of the first complex societies [27,28]. Domestication of animals and plants requires an increasingly routinized, coordinated workforce, which might be achieved through increases in the frequency of communal rituals and homogenization of traditions within a society. In this way, high frequency low-arousal religious systems could facilitate group cooperation and control over resources [27]. These systems are likely to have spread via standardization and codification of language, leading to increased efficiency of broadcasting (i.e. one to many) transmission, resulting in cultural selection between rival groups over longer time spans [4]. On the other hand, small-scale groups that experience food scarcity require extensive sharing of resources to survive [29], and therefore are vulnerable to free-riders. As such, rare and highly arousing rituals bind participants in these groups, and ensure strong commitment to group survival on unproductive and circumscribed lands, where mobility is restricted, making the group more competitive towards out-groups.

Given the complexity of the processes involved on many different social levels and spatial scales, computational and theoretical modelling will be necessary to uncover the key driving processes in the dynamics of human groups, their evolution and diversity. To date, only a limited number of such studies have been conducted [30–36]. Most of these investigations have been very specific and focused on either the characteristics of polities such as information processing, problem solving and adaption, or testing specific theories on state formation or cultural complexity.

Here we develop a spatially explicit, agent-based model as a first step to investigate the population dynamics and socio-cultural evolution of human groups. We have two scientific objectives. First, we aim to make predictions about how the spatial distribution of environmental hospitability (in terms of the number of people who can be supported) affects the emergence of political entities (e.g., nation states) and the rates and effects of warfare. Second, we have the larger objective of proposing a life-cycle theory of human groups, and to see to what extent the dynamics are predictable from parameters and initial conditions. To do this, we draw analogies from the evolution of multicellularity [37]. In our model, each agent represents a sociocultural group resembling a living organism that undergoes birth-death processes. Small-scale, unstructured groups are represented as single-celled units, which live as autonomous entities and have control over their actions, while large-scale, structured groups are akin to multicellular organisms, where each component cell relinquishes its autonomy to some extent to the larger whole [37,38]. There are two main parallels between the adaptive reasons of formation of larger scale societies and multicellularity: to better avoid attacks from other groups/ avoid attacks from predators, and to acquire new, beneficial cultural traits through division of labour and cultural transmission / acquire new specialized cell functions. However, there are costs to maintain a larger group size both in the case of multicellularity and human social groups [37,39]. In both cases, individual units should be able to maintain cooperation through conflict mediation which involves mechanisms such as policing by the immune system or programmed cell death in the case of multicellular organisms [37]; and the establishment of strong institutions in the case of human groups [40].

Our model is designed to have features shared by a range of group types. These groups may refer to hunter-gatherers vs. agrarian groups, political organizations and nation states, and religious groups. Given that many questions remain unanswered about the relative importance of the drivers of social group dynamics (i.e., demography, cultural and social evolution), and that we have empirically-based estimates for only a small number of model parameters, we define flexible rules for group birth, growth, competition and death. These rules are evaluated through sensitivity analyses to assess the importance of their effects, and to see which require further investigation. In particular we systematically examine the effects of (i) resource distribution (i.e. distribution and ratio of productive and unproductive environments), (ii) threshold levels for the fusion of different entities (transition from single-cell to multicellular structure), (iii) population growth rates, and (iv) the factors determining the outcome of competition and warfare.

## Methods

### Basic model features

**Fig 1:**
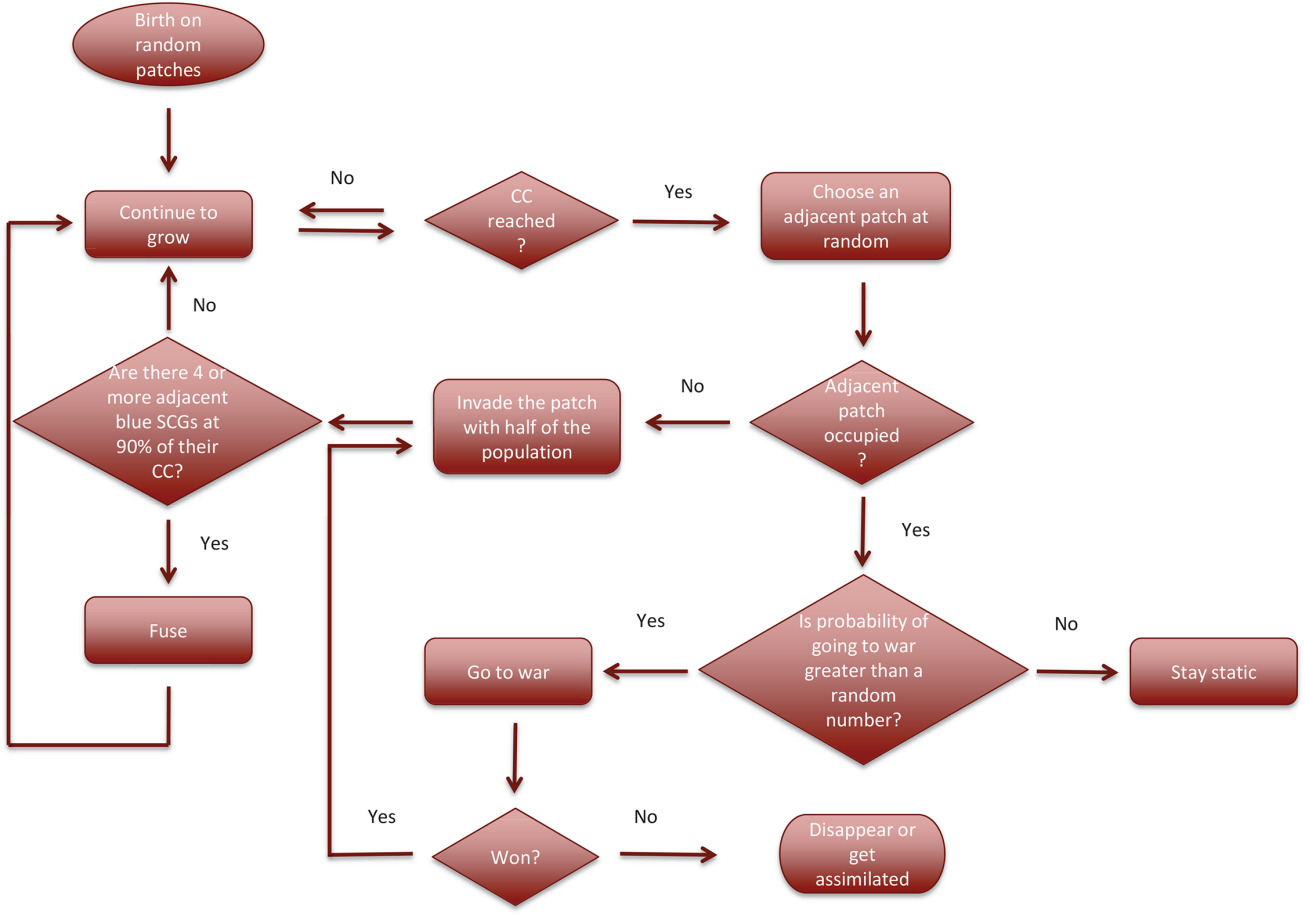
Model flow chart. See main text and S1 Information for detailed description.

The flow diagram for the model is presented in Fig 1, and described below and in more detail in S1 Information. Parameter descriptions and baseline values are given in Table 1.

**Table 1:** Parameters and their baseline values.

Parameter	Value
High-productivity (HP) patch carrying capacity	100
Low-productivity (LP) patch carrying capacity	50
Growth-rate on HP patch	0.03
Growth-rate on LP patch	0.01
Ratio of LP-to-HP patches at *t* = 0	1:1
Initial number of SCGs on HP patches	10
Initial number of SCGs on LP patches	10
Initial population size of SCGs	30
Constant (*c*)	0.001
Cost constant (*c*_*1*_)	1
Threshold for fusion of blue SCGs	4
Carrying capacity threshold for fusion	90%

The system is a square lattice of patches (33 x 33, corresponding to exploitable land) with natural, absorbing edges (i.e., non-reflecting and non-wrap around). Patches each have one of two resource productivity levels (high or low) determining the maximum number of people who can occupy that patch (i.e., the carrying capacity (CC)) and local population growth rates (Table 1). The numbers of low-productivity and high-productivity patches and their distributions can be varied (from random to clustered) to produce different landscapes. We assume that the populations in each patch potentially interact with any of four neighbouring patches.

Each group is characterized by several variables. First, a group is made up of one or more cells, each of the latter occupying a single patch. Single-celled groups (SCG) occupy one patch only, whereas a multicellular group (MCG) occupies 2 or more contiguous patches. Second, each group has a level of aggression (referred to as “redness”) towards neighbouring groups, determined by the productivity of the patches they occupy. This is because, shortage of a productive land in a growing population is one of the main determinants of warfare [9]. Therefore, we denote a SCG on a low-productivity patch as a ‘red SCG’ (akin to warrior groups) with redness level set to 1, and a SCG on a high-productivity patch as a ‘blue SCG’ (peaceful groups) with a redness level at 0. In contrast, a MCG group can occupy terrain that includes both low productivity (LP) and high productivity (HP) patches, in which case, the redness of a MCG is calculated by taking the mean of all component cells. Third, groups on high-productivity patches have higher growth rates and higher carrying capacities than those on low-productivity patches. Fourth, each group has a heritable marker (ID) on a one-dimensional scale from 0 to n, where n is the initial number of groups. Fifth, groups make random decisions about expanding into neighbouring patches.

### Initial Conditions

The landscape is established by assigning a given number of high-productivity and low-productivity patches to x,y coordinates on a square lattice (S1 Fig). The distribution of low and high productivity patches is varied as (1) a random, uniform distribution (S2A Fig), (2) aggregation of at least 9 HP patches (S2B Fig), (3) aggregation of at least 49 HP patches (S2C Fig), or (4) aggregation of at least 81 HP patches (S2D Fig). 10 SCGs are randomly located on HP patches (blue SCGs), and 10 others are randomly located on LP patches (red SCGs). The initial population size of each group is set to 30.

### Numerical methods

**Fig 2:**
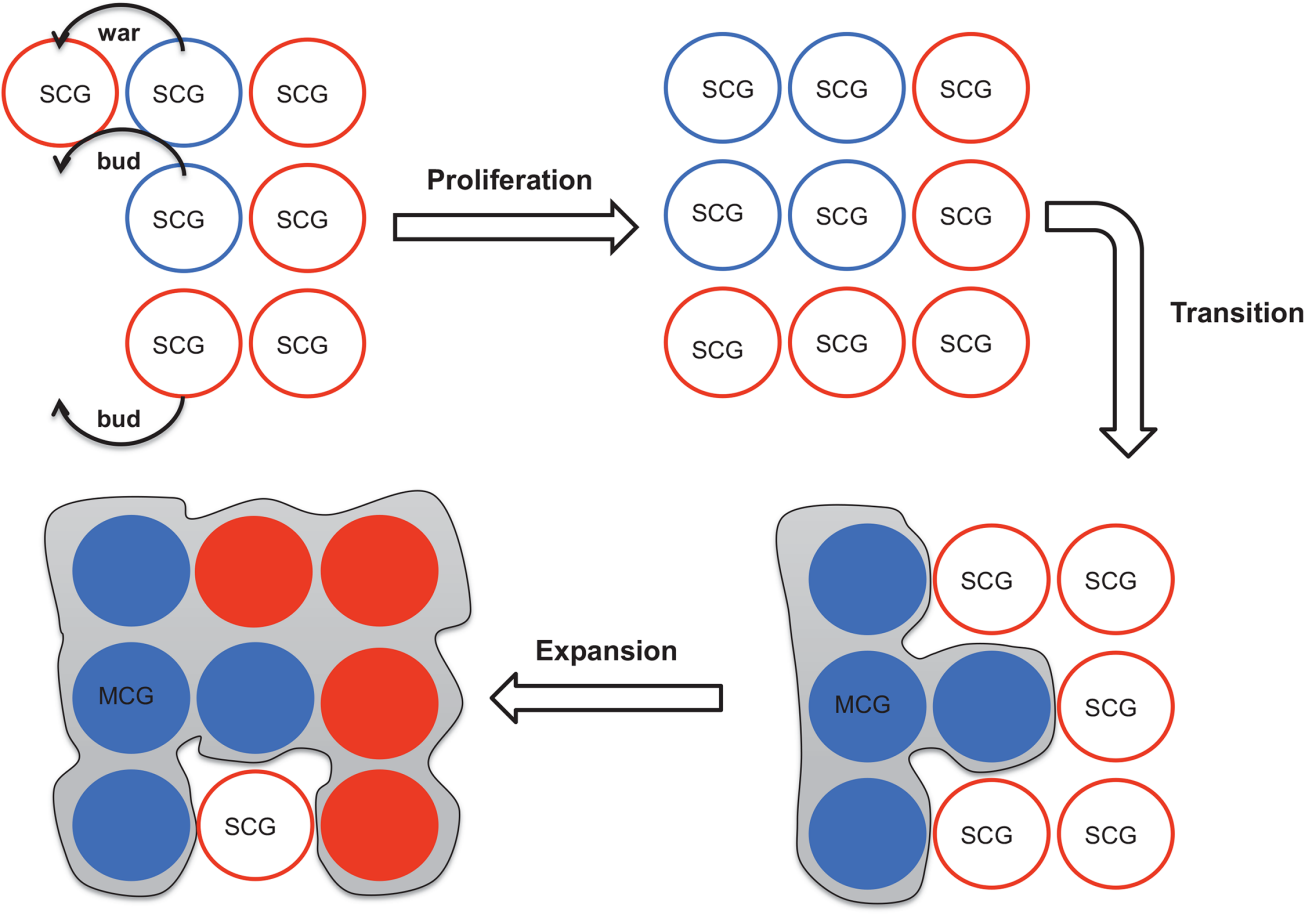
Lifecycle model of human groups. SCG refers to single-celled groups (a representation of small-scale, unstructured human groups); MCG refers to multicellular groups (a representation of large-scale, structured human groups). Red SCGs (SCGs on low-productivity patches) are shown as red circles with white filling, while blue SCGs (SCGs on high-productivity patches) are shown as blue circles with white filling. SCGs proliferate either by budding or going to war with the adjacent groups. Transition from SCGs to a MCG occurs when a number of (default value = 4) adjacent blue SCGs reach to a certain percentage (default value = 90%) of their carrying capacities and fuse. A MCG expands via growing or going to a war with adjacent groups.

Numerical experiments begin after initializing the landscape as described above and in S1 Information (see Table 1 for default values). At each time step, each cell in the system grows unless its population size exceeds its CC. Then, a fixed number of cells (default value = 20) are chosen at random and each makes a decision on whether to spatially expand, go to war, or remain static. If the cell population does not exceed its CC, then it is assumed not to change; otherwise, it either buds, goes to war, or chooses to stay static, depending on surrounding patches (Fig 1). After each decision, if there are ≥4 blue SCGs at >90% of their CCs on the landscape, then one group is chosen as the reference for the possibility of a transition from SCGs to a MCG (Fig 2). If the chosen group has 3 or more adjacent blue SCGs fulfilling the requirement for fusion (i.e. being at >90% of their CC), they all fuse to become a new MCG. When all the chosen cells make their decisions, the cells grow again. The time step between two consecutive growth periods is one year. Each numerical experiment is run for 600 years (see S1 Information for the details). We conducted replicated numerical simulations with the following experimental treatments, varying: (1) productivity patch ratios, (2) the threshold number for fusion of blue SCGs, (3) patch aggregation levels, (4) growth rates, and (5) constant parameters in the war functions. When studying the effects of constant parameter values, the target parameter was varied and all other parameters were kept at their default values (Table 1). We ran 15 replicates of each numerical experiment and examined changes in the numbers and distribution of each group (red SCG, blue SCG and MCG) over time. We calculated the mean numbers of SCGs, MCGs and cells belonging to MCGs in every year and compared these means with those resulting from the default parameter set. In addition, we calculated the mean frequencies of MCGs with respect to their sizes (i.e. the number of component cells) every 100 years and compared the final frequency distributions with the default conditions. All simulations were conducted in NetLogo multi-agent programmable modelling environment [41] and data processing was performed in the R interactive statistical environment [42]. The simulation data for each numerical experiment (Dataset S1 in S1 Information) and key (Data key S1 in S1 Information) are provided with S1 Information.

## Results

### Basic model behaviour

**Fig 3:**
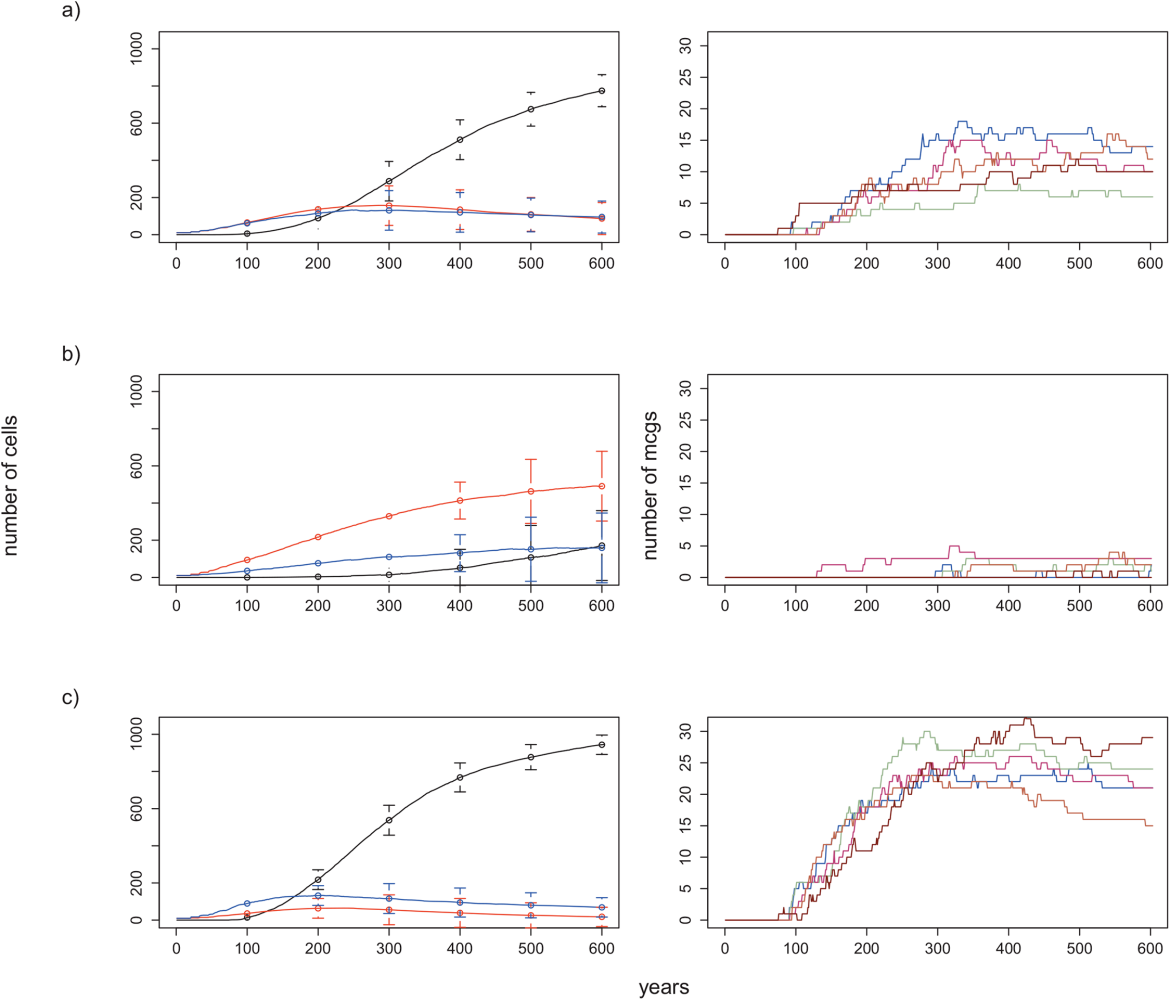
Effect of patch ratio on the number of cells and on the number of MCGs formed over 600 years. Plots on the left: change in the numbers of cells belonging to MCGs (black line), red SCGs (red line) and blue SCGs (blue line) over 600 years. Lines correspond to the mean numbers in 15 replicates and vertical bars the standard deviations for corresponding years. Plots on the right: number of MCGs formed during 5 replicates of a numerical experiment with identical starting conditions. a) LP/HP patch ratio = 1:1 (default condition). b) LP/HP patch ratio = 3:1. c) LP/HP patch ratio = 1:3. All other conditions are the same as the default condition (Table 1).

**Fig 4:**
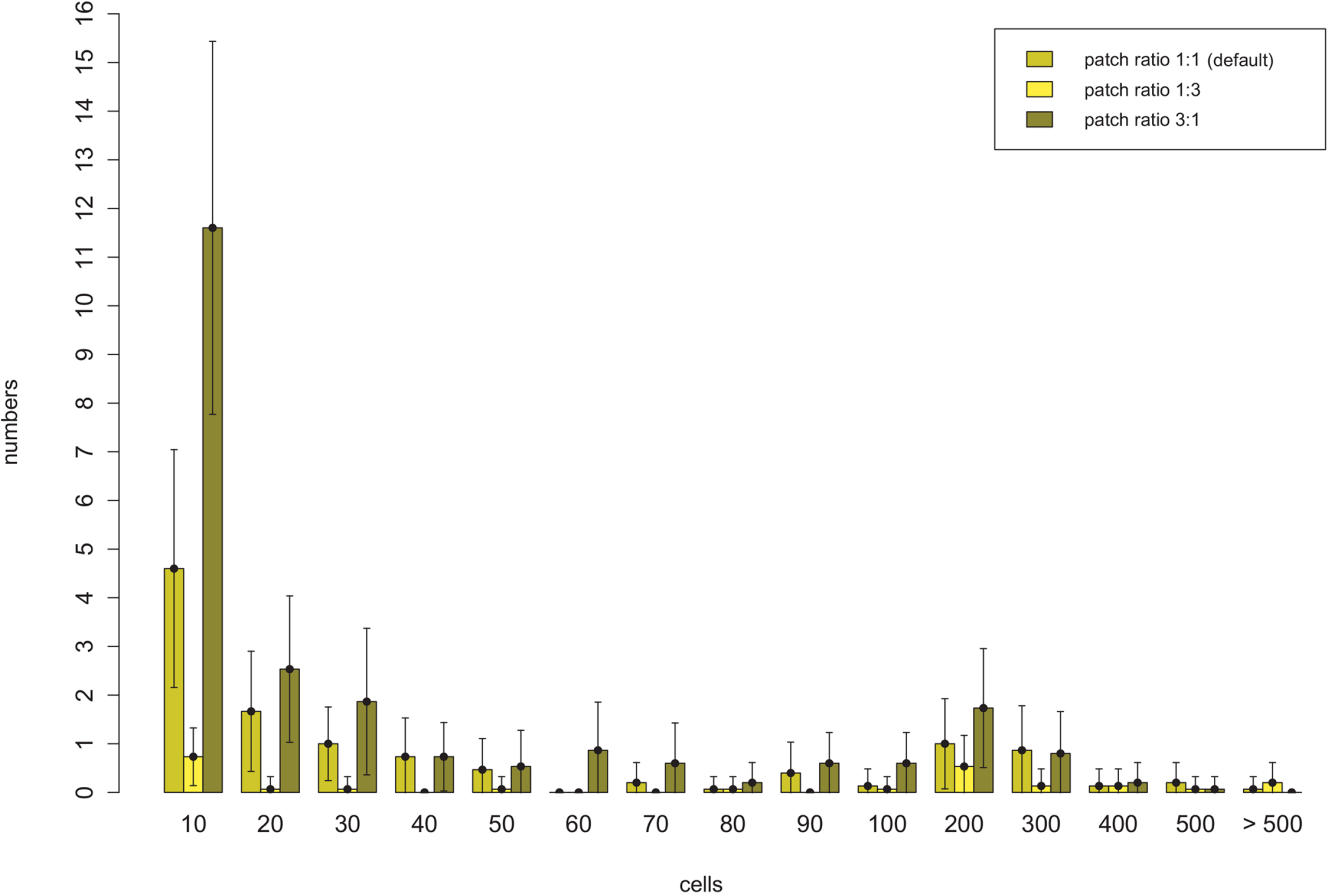
Effect of patch ratio on group size distribution at *t* = 600. The bars show frequencies of MCGs with respect to the number of their component cells averaged over 15 replicates. Light green bars represent frequencies for the experiments where the HP/LP patch ratio = 1:1. Yellow bars represent those where HP/LP patch ratio = 1:3 and dark green bars those where HP/LP patch ratio = 3:1. 10 indicates the numbers of cells between 2 to 10, 20: 11 to 20, 30: 21 to 30, …, 200: 101 to 200, 300: 201 to 300, …, > 500: more than 500. Vertical bars are standard deviations.

Under the baseline conditions with an initial random patch distribution, MCGs emerged after about 100 years and the cells belonging to MCGs continued increasing in number, whereas SCGs initially increased in number, attaining constant populations before finally declining (Fig 3A). After their establishment, the number of MCGs increased for the first 200 to 300 years, and then attained more or less constant numbers (Fig 3A, right caption). The number of MCGs eventually decreased as the number of constituent cells continued to increase, such that the majority had between 2 and 10 cells at 600 years. Interestingly, for many of the replicates, MCGs of between 50 and 100 cells were either absent or low in numbers compared to those with lower (2 to 50) or higher (100 to 500) cell numbers (Fig 4, S3 Fig), indicative of a conserved divergence in growth rates. In many simulations, a number of MCGs reached more than 100 constituent cells and began to dominate the landscape after about 300 years (S4A Fig). Based on our numerical experiments we suggest that given enough time, a single MCG will eventually come to dominate more than 50% of the landscape. For instance, in a numerical experiment that lasted for more than 1500 years, a single MCG dominated the landscape by occupying 69% of the area at the end of 1400 years (S5A Fig). The same experiment also revealed that the dominant MCG could decrease substantially in size due to war, but soon recover its losses (S5B and S5C Fig). The dominance of a single MCG was, not surprisingly, associated with overall fewer MCGs after about 1000 years (S4D Fig).

### Low-productivity/high-productivity patch ratio

**Fig 5:**
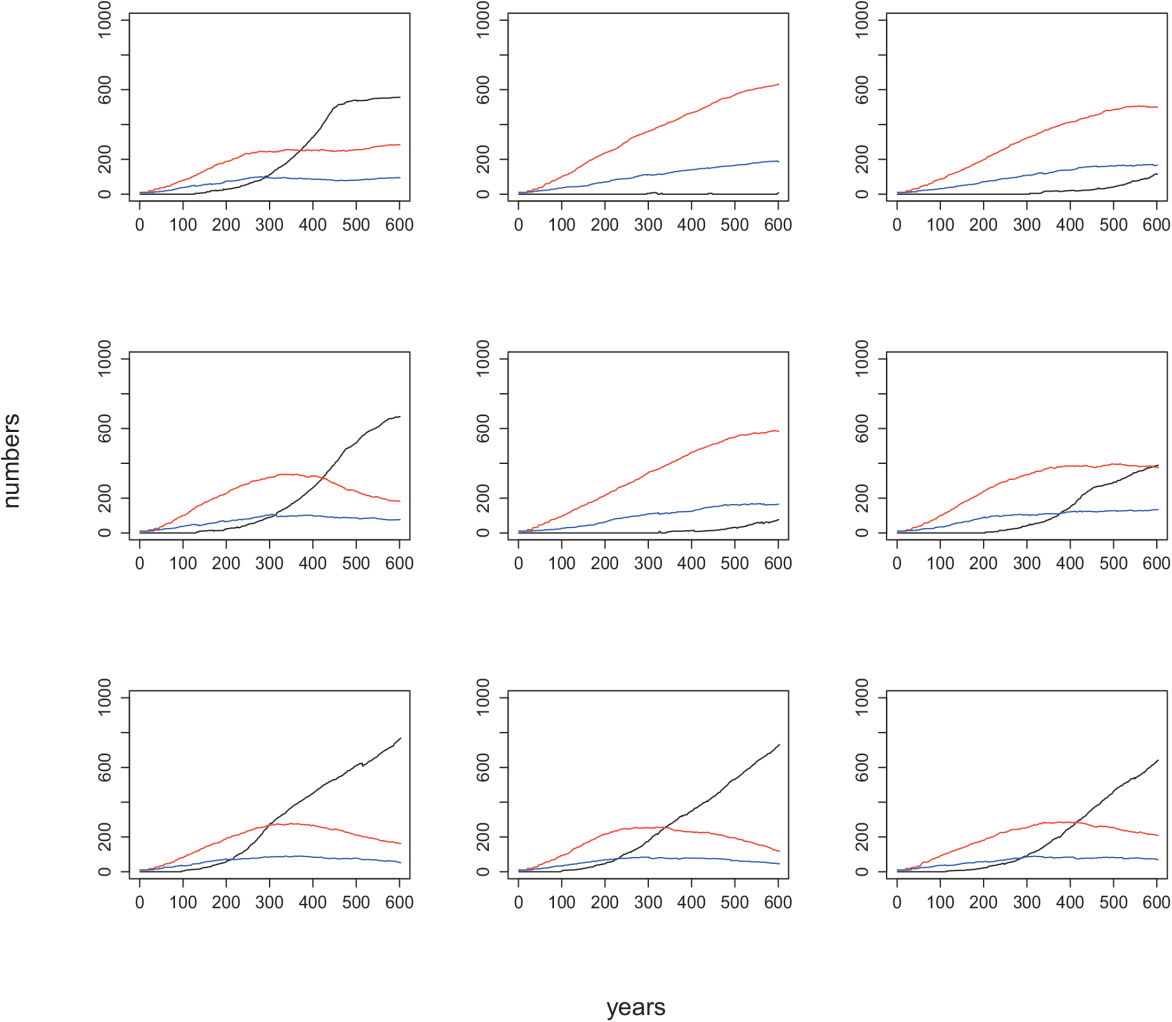
Change in the number of cells belonging to MCGs (black line), red SCGs (red line) and blue SCGs (blue line) over 600 years in 9 replicates of a numerical experiment with high LP/ HP patch ratio. The initial conditions are the same as the default condition, except for the LP/HP patch ratio = 3:1.

When the LP/HP patch ratio is set to 3:1, the required time for the first MCG to form increased, because of its dependence on the (lower) probability of observing 4 or more adjacent blue SCGs. Moreover, once formed, MCGs require suitable conditions for their expansion on the landscape. For instance, if a newly formed MCG is surrounded by red SCGs, then its chance to spread over the landscape decreases drastically. In fact, we observed that many MCGs perished a few years after they formed, since they could not resist the attacks of surrounding red SCGs. As a result, mean MCG cell numbers and their total group numbers after 600 years were lower than observed for the other patch ratios (Figs 3B and 5). However, the variation in the number of component cells of MCGs among replicates was higher than that observed for patch ratios with larger proportions of high productivity patches (see the error bars: Fig 3B and Fig 5). This is because MCG formation is less probable in low productivity landscapes, and a small number of these groups come to dominate (Figs 3B and 5 and S6 Fig). Consistently, by *t* = 600, MCGs had either <10 cells or >200 cells (Fig 4, S4B Fig).

When the landscape was dominated by high productivity patches (LP/HP = 1:3), after their emergence at around *t* = 100, cells belonging to MCGs increased in number more steeply than for the baseline patch ratio of 1:1 (Fig 3C). To see how landscape influenced MCG dynamics, we conducted a one-way ANOVA, which showed that the total number of MCGs formed at the end of 600 years was significantly higher for 1:3 compared to the two other patch ratios considered (*p<* 0.001; Figs 3C (right caption) and 4). The frequency distribution of MCGs with respect to the number of component cells had a more dispersed pattern with relatively more medium-size MCGs compared to the experiments with 1:1 or 3:1 ratios (Fig 4, S4C Fig). Like for the patch ratio of 1:1, there was more small cell number MCGs (between 2 and 10) than large cell number MCGs.

### Threshold for the birth of MCGs

**Fig 6:**
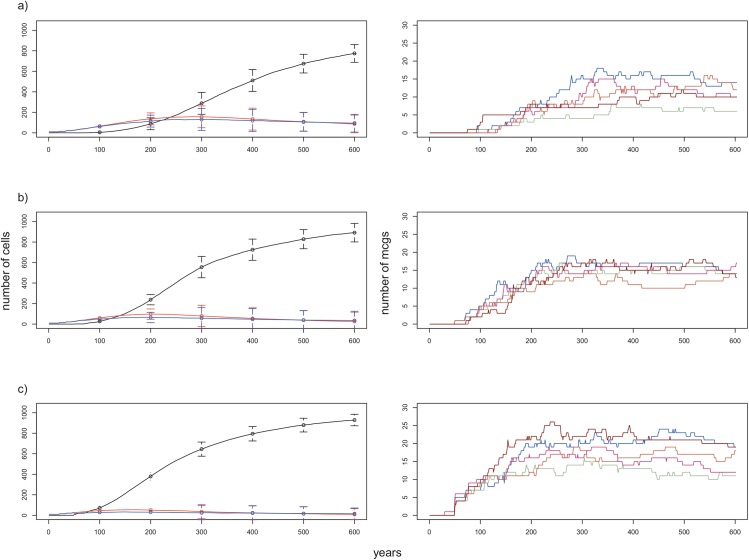
Effect of threshold for birth of MCGs (i.e. fusion of blue SCGs) on the number of cells and on the number of MCGs formed over 600 years. When the threshold is lowered, MCGs start to form earlier and reach to higher numbers. Plots on the left: change in the numbers of cells belonging to MCGs (black line), red SCGs (red line) and blue SCGs (blue line) over 600 years. Lines correspond to the mean numbers in 15 replicates and vertical bars are standard deviations for corresponding years. Plots on the right: number of MCGs formed during 5 replicates of an experiment. a) Threshold for fusion = 4 (default condition). b) Threshold for fusion = 3. c) Threshold for fusion = 2. All other conditions are the same as the baseline conditions.

As expected, when the threshold number for the fusion of blue SCGs was lowered below 4, MCGs began to form earlier. In all cases, MCG cells continued increasing in number while SCGs initially increased and then attained constant populations before finally declining (Fig 6, left caption). Moreover, in all experiments MCGs increased in number for a few hundred years until they reached more or less constant numbers (Fig 6, right caption). The number of MCGs formed over the first 600 years was significantly different among the three different threshold values (*M* ± SD for 4: 7.92 ± 1.86, for 3: 12.12 ± 1.99, for 2: 14.77 ± 2.04, one-way ANOVA: *p<* 0.001). The number of MCGs after 600 years was also significantly higher than for the baseline condition (one-way ANOVA followed by Tukey’s HSD post-hoc test: *p<* 0.01 for both 2 and 3; n.s. between 2 and 3). The pattern in the mean frequency distribution of MCGs with respect to their constituent cells was the same as the default condition (S7 Fig). For both threshold values, the number of MCGs having less than 10 cells at *t* = 600 was significantly higher than the number for the baseline condition (one-way ANOVA followed by Tukey’s HSD post-hoc test: *p<* 0.05 for 3 and 2). In general, a small number of MCGs with more than 200 cells dominated the landscape at *t* = 600 (S8 Fig).

### Patch aggregation

**Fig 7:**
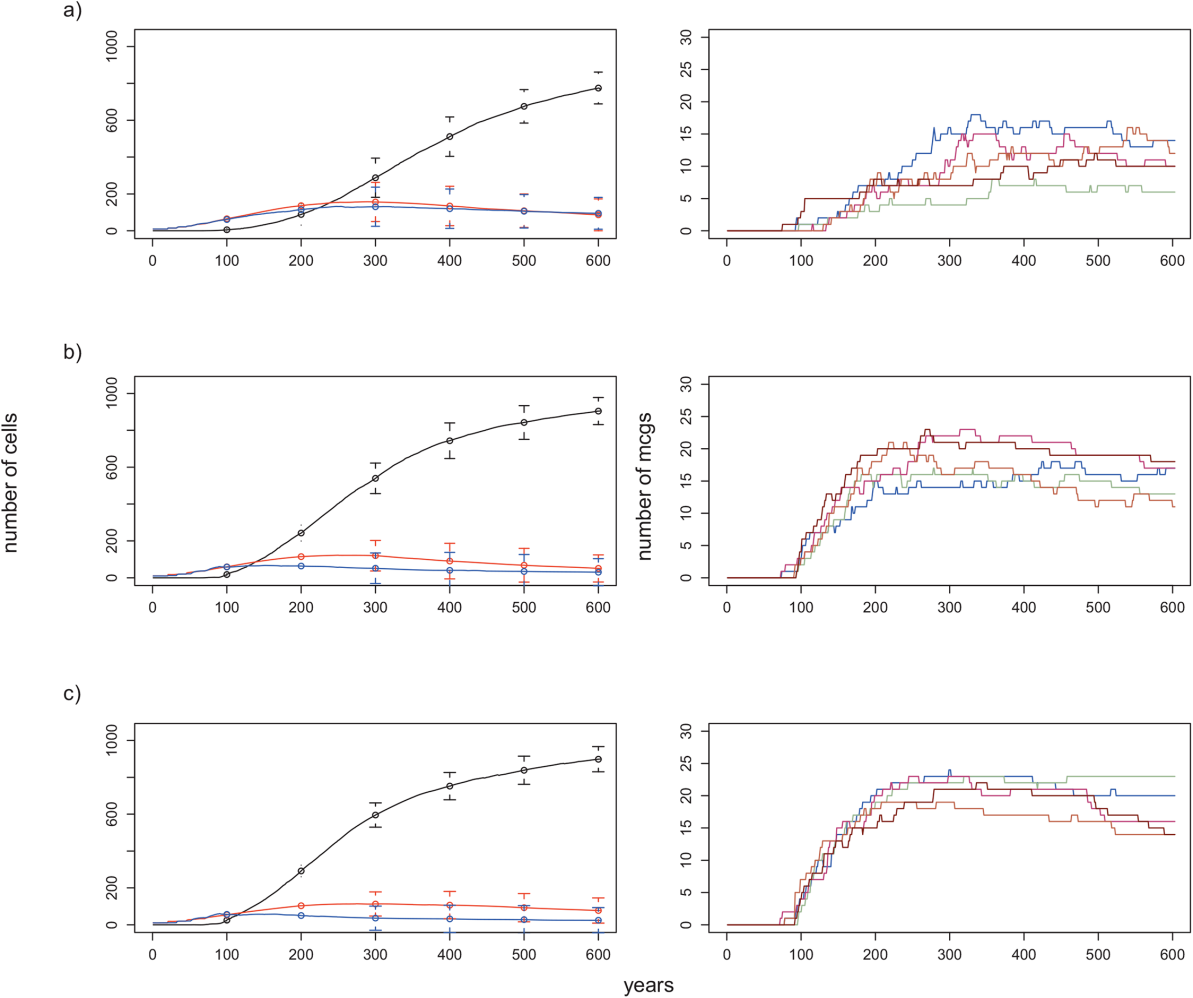
Effect of patch aggregation on the number of cells and on the number of MCGs formed over 600 years. Significantly more MCGs are formed when the patches are aggregated. Plots on the left: change in the numbers of cells belonging to MCGs (black line), red SCGs (red line) and blue SCGs (blue line) over 600 years. Lines correspond to the mean numbers in 15 replicates and vertical bars mark standard deviations for corresponding years. Plots on the right: number of MCGs formed during 5 replicates of an experiment. a) LP and HP patches are randomly distributed (default condition). b) At least 49 HP patches are aggregated. c) At least 81 HP patches are aggregated. Other conditions are the same as the default condition.

Aggregation in patch productivity had little effect on MCG emergence (Fig 7). However, once formed, MCG cells increased more rapidly compared to the baseline condition (Fig 7 left caption). For all three levels of aggregation (at least 9, 49 or 81 HP patches in clusters), the number of MCGs formed over the first 600 years was significantly higher than for the baseline condition (one-way ANOVA followed by Tukey’s HSD post-hoc test: *p<* 0.001), although there was no change in the frequency distribution of MCGs at *t* = 600, nor differences in the frequencies of MCGs for different numbers of constituent cells among different patch aggregation levels (S9 and S10 Figs).

### Probabilities of going to war and victory, and cost equations

**Fig 8:**
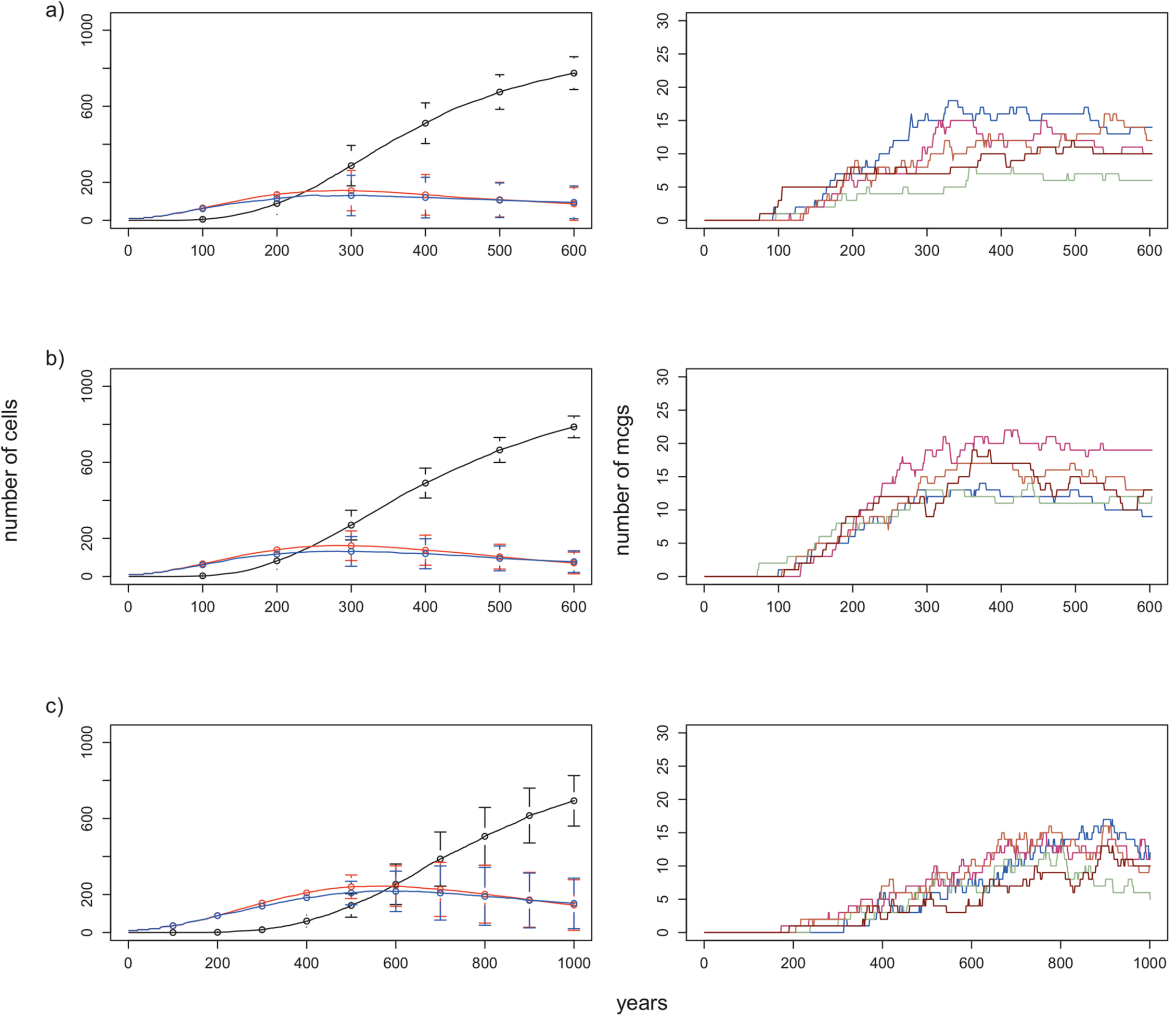
Effect of constant for cost functions and growth rate on the number of cells and on the number of MCGs formed over 600 years. Plots on the left: change in the numbers of cells belonging to MCGs (black line), red SCGs (red line) and blue SCGs (blue line) over 600 years. Lines correspond to the mean numbers in 15 replicates and vertical bars mark standard deviations for corresponding years. Plots on the right: number of MCGs formed during 5 replicates of an experiment. a) Growth rate on LP patches = 0.01, on HP patches = 0.03, constant (*c*) = 0.001 (default condition). b) Growth rate on LP patches = 0.01, on HP patches = 0.03, constant (*c*) = 0.1. c) Growth rate on LP patches = 0.005, growth rate on HP patches = 0.01, constant (*c*) = 0.001. All other initial conditions are the same as the default conditions.

The constant *c* employed in the warfare functions determined the relative importance of population size to this type of event. For instance, when *c* = 0.001, the population size of a group did not have much impact, especially when group redness was 0. Increasing the value to 0.1 had little effect on the number of component MCG and SCG cells (Fig 8A and 8B). However, we found that the actual number of MCGs significantly increased (one-way ANOVA: *p<* 0.001), although the frequency distribution did not change (S11 (red bars) and S12B Figs).

### Growth rate

There is some controversy concerning the per annum growth rate of past hunter-gatherer populations, some authors arguing that hunter-gatherer groups never attained rates exceeding 1% per year [43]. To investigate this, we lowered the growth rate for HP patches to 0.01 and for LP patches from 0.01 to 0.005, and found, as expected, that cells took longer to reach their carrying capacities. As a result, MCGs began to form later than observed in the baseline parameter set, and the number of cells belonging to MCGs continued increasing, while SCGs increased in numbers for 500 years and then began to decrease (Fig 8C). As expected, the number of MCGs formed over 600 years was significantly lower than that for the baseline condition (one-way ANOVA: *p<* 0.01), however there was no significant difference between the numbers of MCGs at the end of 1000 versus 600 years (one-way ANOVA: *p* = 0.87, Fig 8A–8C, S10 Fig (blue bars)). At t = 1000, the number and frequency distribution of MCGs was the same as for the baseline condition (S11 (blue bars) and S12C Figs).

## Discussion

We used the analogy between small vs. large-scale human groups and single vs multi-cellular organisms to develop a theory of group lifecycles as an emergent property of single cell demographic and expansion behaviours (Fig 2). Our results are in agreement with the broad tendency of a small number of nations becoming much larger in size than the majority of nations [15,44,45]. Specifically, we found that the transition from single-celled groups (SCGs) to multi-cellular ones (MCGs) takes about 100 simulation years, but the timing also depends on the threshold for the birth of MCGs and the growth rate of local populations or cells. Once the transition occurs, the fused cells—MCGs—spread rapidly and dominate other groups, mainly by outnumbering them in warfare. Although many of the demographic and expansion behaviours followed logically from model assumptions, the complexity of the system led to some unexpected results. Most interesting among them is that although gradually dominating SCGs, MCGs diverge in polity size, resulting in a U-shaped distribution that persists over hundreds of years. We assumed a linear relation between group size and the probability of warfare victory [46–48]; had we assumed a squared law, which is more realistic for modern warfare [48], we predict that the U-shaped distribution would still emerge, but that it would emerge faster and be more skewed to the larger groups. Future studies should explore alternative assumptions about warfare.

Our sensitivity analysis revealed that the ratio and the distribution of patches with high and low productivity levels play crucial roles in the trajectory of sociocultural evolution (i.e. change in the numbers of small-scale and large-scale groups). Archaeological data suggests that complex societies could only be formed on agricultural lands [9].It is generally accepted that agriculture enabled the maintenance of high population densities that eventually lead to complex societies [9,12,14]. In support, we observed significantly lower numbers of MCGs when the landscape was largely composed of unproductive land (i.e. LP/HP patch ratio: 3:1). This result was expected for two reasons: 1) the reduced chance for required number of blue SCGs to come together and fuse, and 2) given our assumption that groups on unproductive lands are more aggressive[24], many of the blue SCGs that came together to become a MCG immediately got defeated by the attacks of surrounding red SCGs. Importantly, we also observed considerable variation among replicates for the same experiment (e.g., Fig 3B (error bars), Fig 5), suggesting that for certain initial conditions, subsequent stochasticity in events could change the trajectory of history each time we conducted a numerical experiment.

Increasing the numbers of productive patches or initializing the landscape with an aggregated patch distribution did not have a major effect on MCG emergence times. Roughly speaking, for all levels of patch aggregation, MCGs began to form after about 100 years. This suggests that it was only after this same time that many SCGs reached 90% of their CC. Obviously we would expect an earlier emergence of MCGs if the criterion for fusion were lowered to less than 90% of CC. However, according to theories of state formation, complex large-scale groups only begin to form in the presence of population pressure following their growth and proliferation [9,49]. Furthermore, establishment of necessary norms for cooperation and coordination is a relatively slow process during the emergence of complex groups [14]. When we lowered the threshold number for fusion from 4 to 3, and then to 2, MCGs began to emerge earlier (Fig 6). Both lower threshold values for fusion, greater patch aggregation levels, and greater HP/LP patch ratios resulted in higher numbers of MCGs, since all these conditions favoured the initial establishment of MCGs, leading to greater transient MCG diversity.

**Fig 9:**
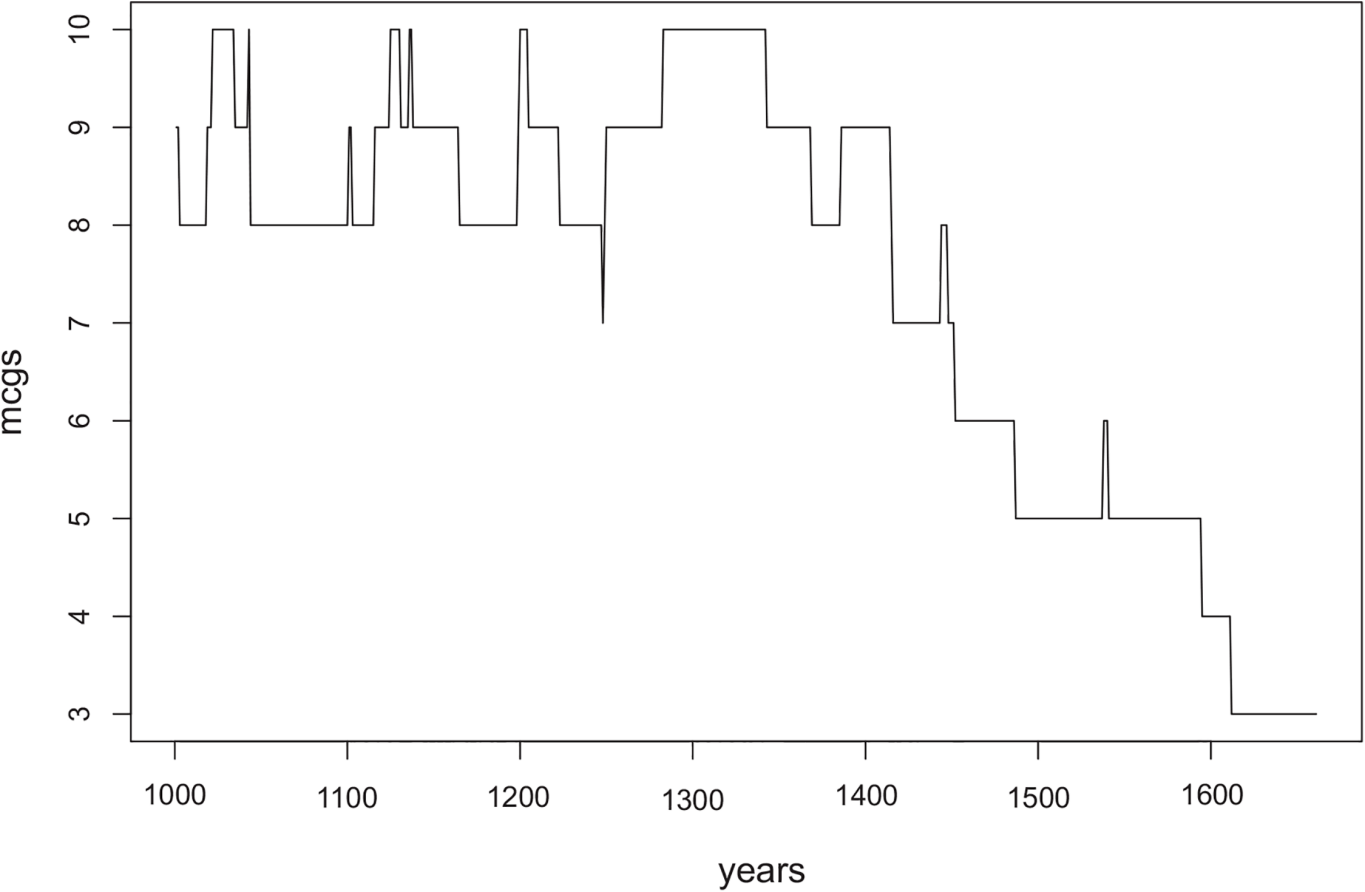
Decrease in the number of MCGs after *t* = 1000 in our simulation with the default condition that lasted 1660 years. From *t* = 1000 to around *t* = 1300 MCGs fluctuate around the same numbers, however during the last 330 simulation years the number of MCGs decreases exponentially until only one MCG dominates the whole landscape.

The phenomena observed in our simulations are in agreement with observed trends in the increase of polity size and decrease in the number of separate political entities over 5000 years after the formation of the first state [44]. For instance, between 3000 BCE and 600 BCE the largest polity (most often centred in Egypt) occupied 0.3–1% of the dry land area. From 600 BCE to 1600 CE, the largest sizes gradually increased from 5 to 20% of dry land area (examples to these large polities: Achaemenid Persia and Genghis Khan’s empire). Around 1600 CE, another sharp increase in size occurred giving rise to polities such as British Empire and Soviet Russia occupying even 25% of dry land area [15,44]. While the trend was to increase in polity size, the number of autonomous political units in the world has decreased from approximately 600,000 in 1500 CE to just fewer than 200 today [45]. In conjunction with the results of our simulations, this decline is shown to be exponential (cf. Fig 9 with Fig 5 in [44]) and by fitting an exponential model on the historical data regarding the areas of the largest polities, it has been predicted that there is a 50% probability that the world will be a single polity by 3800 CE. Another study [45] predicts this date to be 2300 CE by plotting the decline in number of autonomous political units from 1550 B.C to the present. Our model goes beyond these previous statistical models by providing simple mechanisms for the emergence, growth and dominance of states.

Despite the trend in history towards larger and fewer polities, there is a restriction on the growth of human groups due to “scalar stress”, whereby the ability of leaders to process information and control over subordinates decreases as society increase in number and spatial occupation [50]. This is very similar to what we observe in the formation of multicellular organisms: the cost of reproduction increases with increasing group sizes, because the increase in fecundity in larger groups is associated with decrease in viability [37]. The cells overcome this problem by undergoing germ and soma specialization [37]. In case of human groups, many scholars have argued that in order to overcome the costs of expansion, a group needs to establish a hierarchical structure and necessary institutions, whereby each superior directly controls a certain number of subordinates [8,9,15,36,50,51]. Leaders of expanding societies that lack this elaboration lose their abilities to process information and control subordinates, eventually leading to fragmentation or collapse of the group [17,50,52]. Recently, by using a simulation model researchers demonstrated that ultrasocial norms and institutions selected as a result of intense warfare could explain the 65% of the observed variance in the history of Afroeurasian large-scale societies [36].

Correspondingly, in our simulations we observed that some cells of a MCG became detached from their group leading to spatial fragmentation into two or more pieces as a result of warfare. In the current model even when a MCG broke into two or more distinct aggregates, all were assumed to continue to be the part of the previous group. Future models should explicitly include a cost of reproduction associated with the group size of an MCG, and probabilities of MCG fragmentation due to scalar stress. The splintered parts whether occurring via war (as assumed here) or through internally driven fragmentation (e.g. lack of strong decision-making), then might either mutate their cultural identity during the separation or acquire a new one in a process similar to the fixation of a neutral marker in isolated populations. We predict that this birth process and subsequent diversification would affect the long-term trajectory of the system, and change the U-shaped frequency distribution of MCGs.

Here, we introduced a simple approach to study the dynamics of human groups by analysing group life cycles as an emergent property of single cell demographic and expansion behaviors. The flexibility of our algorithm allows for many possible lines of investigation and enhancement, including the effects of resource depletion, continuous variation in landscape productivity levels, and how incorporating rational decision-making may affect the persistence of different groups.

## Supporting Information

S1 Information Detailed description of the model. (DOCX)

S1 Fig Example of a snapshot from the simulation interface in NetLogo in year 238. Neighboring patches of a MCG are shown as yellow and that of a SCG as orange. Low-productivity patches are shown as brown squares and high-productivity patches as green squares. SCGs are represented as blue or red circles depending on the productivity of the patch they occupy. The circles with black filling are part of MCGs. Each group has an ID number as a label. Each MCG has a unique label color.
(TIF)

S2 Fig Alternative patch distributions at *t* = 0. a) HP and LP patches are randomly distributed. b) Aggregation of at least 9 HP patches. c) Aggregation of at least 49 HP patches. d) Aggregation of at least 81 HP patches.
(TIF)

S3 Fig Frequency of MCGs at *t* = 600 with respect to the number of component cells in 15 replicates of a numerical experiment with the default condition. 10 indicates the numbers between 2 to 10, 20: 11 to 20, 30: 21 to 30, …, 200: 101 to 200, 300: 201 to 300 and so on. Note that the replicate shown by the plot on the top left side had a MCG that dominated the landscape with 836 component cells (occupying 77% of the landscape). In general, many MCGs had less than 20 cells and a few others expanded on the landscape with more than 100 cells.
(TIF)

S4 Fig Mean frequency of MCGs with respect to the number of their component cells during different time intervals of 3 numerical experiments with different patch ratios. The black bars show the frequencies at t = 600. a) LP/HP patch ratio = 1:1 (default conditions). b) LP/HP patch ratio = 3:1. c) LP/HP patch ratio = 1:3.
(TIF)

S5 Fig A numerical experiment with the default initial condition that lasted 1660 years. a) A snapshot from NetLogo at *t* = 1400. The MCG with ID number = 18 (MCG-18) occupies 69% of the landscape with 756 constituent cells. b) A snapshot at *t* = 1600 shows that MCG-18 is more spread over the landscape, however it has also lost some of its cells (mainly the cells on LP patches) over 200 years. c) Change in the numbers of cells belonging to MCGs (black line), red SCGs (red line) and blue SCGs (blue line) over 1660 years reveals the sudden decrease in the number of cells belonging to MCG-18 at *t* = 1570 (this is because MCG-18 lost 394 constituent cells due to a war, but rapidly recovered, reaching to the 1012 cells, occupying 93% of the landscape at the end of 1660 years). d) Change in the number of MCGs over the 1660 years of the simulation.
(TIF)

S6 Fig Two snapshots from NetLogo where a MCG came to dominate the landscape after more than 1000 years. The initial conditions are the same as the default condition, except for the LP/HP patch ratio = 3:1. a) At *t* = 1219 the MCG with the black ID color (ID number = 15) begins to dominate the landscape. The snapshot is taken just after a war between the MCG with the ID = 15 and the MCG with the ID = 10. Sharp decreases in the black line (cell counts) indicate the loss of the cells belonging to MCGs after two consecutive wars. b) At *t* = 1291, the MCG with the ID = 10 perishes and MCG with the ID = 15 continues expanding.
(TIF)

S7 Fig Effect of threshold for birth of MCGs on group size distribution at *t* = 600. The bars show frequencies of MCGs with respect to the number of their component cells averaged over 15 replicates. Dark blue bars represent frequencies for the experiments where threshold for fusion = 4 (default condition). Bars in the middle represent those with the threshold of 3 and light blue bars those with the threshold of 2. 10 indicates the numbers of cells between 2 to 10, 20: 11 to 20, 30: 21 to 30, …, 200: 101 to 200, 300: 201 to 300, …, > 500: more than 500. Vertical bars mark standard deviations.
(TIF)

S8 Fig Mean frequency of MCGs with respect to the number of their component cells during different time intervals of 3 numerical experiments with different threshold levels. The black bars show the frequencies at *t* = 600. a) Threshold for fusion = 4 (default condition). b) Threshold for fusion = 3. c) Threshold for fusion = 2.
(TIF)

S9 Fig Effect of patch aggregation on group size distribution at *t* = 600. The bars show frequencies of MCGs with respect to the number of their component cells averaged over 15 replicates. The turquoise bar represents mean frequencies for the experiments where LP and HP patches are randomly distributed (default condition). The red bars represent different levels of aggregation; at least 9, 49 and 81 HP patches in clusters from left to right respectively. 10 indicates the numbers of cells between 2 to 10, 20: 11 to 20, 30: 21 to 30, …, 200: 101 to 200, 300: 201 to 300, …, > 500: more than 500. Vertical bars mark standard deviations.
(TIF)

S10 Fig Mean frequency of MCGs with respect to the number of their component cells during different time intervals of 3 numerical experiments with different patch aggregation levels. The black bars show the frequencies at *t* = 600. a) LP and HP patches are randomly distributed (default condition). b) at least 49 HP patches are aggregated. c) at least 81 HP patches are aggregated. Other conditions are the same as the default condition.
(TIF)

S11 Fig Effect of constant value for the functions related with warfare and growth rate on group size distribution at *t* = 600 (for red and turquoise bars) and at *t* = 1000 (for blue bars). The bars show frequencies of MCGs with respect to the number of their component cells averaged over 15 replicates. Turquoise bars represent mean frequencies for the default condition. Red bars represent the experiments where constant (*c*) was 0.1. Blue bars represent the condition where HP patch growth rate was set to 0.01 and LP patch growth rate was 0.005. 10 indicates the numbers of cells between 2 to 10, 20: 11 to 20, 30: 21 to 30, …, 200: 101 to 200, 300: 201 to 300, …, > 500: more than 500. Vertical bars mark standard deviations.
(TIF)

S12 Fig Mean frequency of MCGs with respect to the number of their component cells during different time intervals of 3 numerical experiments. The black bars show the frequencies at *t* = 600. a) Growth rate on LP patches = 0.01, on HP patches = 0.03, constant (*c*) = 0.001 (default condition). b) Growth rate on LP patches = 0.01, on HP patches = 0.03, constant (*c*) = 0.1. c) Growth rate on LP patches = 0.005, growth rate on HP patches = 0.01, constant (*c*) = 0.001. All other initial conditions are the same as the default conditions.
(TIF)
