# Supplementary material for: A Life-Cycle Model of Human Social Groups Produces a U-Shaped Distribution in Group Size
Source: PLoS One. 2015 Sep 18;10(9):e0138496. doi: 10.1371/journal.pone.0138496 (PMC4575040; doi:10.1371/journal.pone.0138496)
Supplement: S1 Information — (DOCX) [file pone.0138496.s001.docx]

# Supporting Information

## Model transitions (Fig. 1)

## Cell Growth

If a cell is below its carrying capacity (CC), then it increases in size based on its growth rate at each time step. As a default, the CC is set to 100 for high-productivity patches, and to 50 for low-productivity patches (following data on Amazonian tribes [1]). The default values for the growth rate are: 0.03 and 0.01 for the cells on high-productivity and low-productivity patches, respectively [2].

## Group expansion

If a cell is at its CC, then the group chooses at random a neighbouring patch in which to potentially expand. If the chosen patch is unoccupied, then in the case of SCGs expanding, they transfer half of their population to the new patch and produce a new SCG (i.e. they proliferate). For expanding MCGs, the chosen cell within a MCG transfers half of its population to the new patch. For all budding events, progeny groups have the same ID as the mother group. Therefore, the ID diversity does not increase once a simulation starts; it either stays the same or decreases.

## War

If during group expansion the chosen patch is occupied by another group, then the two groups may go to war. The probability of going to war is assumed to increase with the redness score and the population size of the focal (expanding) group. Our assumption about the redness score is based on the idea that the relative fertility of the landscape is a simple proxy for the relative intensity of resource competition. However, while competition between groups is often associated with limited subsistence resources, conflict could also arise for access to oil, marriageable women, precious metals, political dominance, etc. [3]. For simplicity, we assume that warfare in our model is to gain access to limited subsistence resources (e.g., water and food). Moreover, according to the modes theory of religiosity low-frequency, high-arousal rituals should be more prevalent in groups that rely more on foraging and less on agriculture, since an increased likelihood of food scarcity requires sharing resources and is vulnerable to free-riders. As such, rare and highly arousing rituals (e.g. painful initiation rites, scarification, tattooing, removal of body parts, laceration or insertion of bones or other sharp objects through sensitive tissue) bind participants together in highly cohesive groups and ensure their commitment to the group [4,5]. As a result, these imagistic rituals contribute to strong commitment to group survival on unproductive lands, making the group more competitive towards out-groups. Accordingly, the probability of going to war is calculated as follows:

$$p_{going to war}= \frac{{(c+ R}) \times N}{CC}$$

where *c*: constant, *R*: redness of the focal group (for SCGs 0 or 1 depending on the patch productivity; for MCGs, it is the mean redness of all component cells in the group), *N*: population size, *CC*: carrying capacity. The population size and *CC* of a given MCG are the respective sums of all component cells.

We assume that the probability of victory depends on the relative redness and population sizes of both groups, such that

$${p(A)}_{victory}= \frac{{(c+ R}_{A}) \times N_{A}}{{(c+ R}_{A}) \times N_{A}+ {(c+ R}_{B}) \times N_{B}}$$

where *R_A_*: redness of group A (focal group), *N_A_*: population size of group A, *R_B_*: redness of group B (rival group), *N_B_*: population size of group B. Note that this expression follows Lanchester’s linear law, which is a reasonable approximation for one-on-one combat [6,7]. Future models should consider scenarios in which realistic alternatives such as internal aggregative behaviour leads to Lanchester’s squared law [6–8].

The cost of war is the proportion of the population that is killed in battle. It depends on the relative redness and the population size of the enemy. Cost is calculated as

$$cost(A)=c_{1} \times\left( \frac{\left( R_{B}+c \right) \times N_{B}}{\left( R_{B}+c \right) \times N_{B}+ \left( R_{A}+c \right) \times N_{A}} \right)$$

where *c_1_:* cost constant, *R_B_*: redness of the enemy, *R_A_*: redness of self, *c*: constant, *N_B_:* population size of the enemy, *N_A_:* population size of self (for MCGs, the total population size).

Following war, the winning group occupies one patch of the loser by transferring half of its population from the warring cell, and assimilates the survivors. If the loser is a MCG, its adjacent cell to the winner is occupied and assimilated. If there is only one cell remaining in a MCG following war, then the cell becomes SCG again. If there is only one individual left in a given cell, then cell is assumed to perish.

## Fusion

We assume that if there are 4 or more adjacent blue SCGs that are each at >90% of their CCs, then they can unite and form a MCG (see Fig. 2 for group expansion and fusion). Fusion reflects the expansions of groups beyond a few hundred individuals, a threshold where group members need hierarchical organization to maintain cooperation and coordination to maintain cohesion [9,10]. The ID of the new MCG is set as the ID of the component SCG with the highest population size. If two or more SCGs have the same (highest) population size, then one is chosen at random for ID assignment.

# References

1. Carneiro RL. A Theory of the Origin of the State. Science (80- ). 1970;169: 733–738.

2. Gurven M, Kaplan H. Longevity Among Hunter-Gatherers: A Cross-Cultural Examination. Popul Dev Rev. 2007;33: 321–365.

3. Gat A. Mind the Gap Tracing the Origins of Human Universals. Kappeler PM, Silk JB, editors. Media. Springer; 2010. pp. 197–220.

4. Whitehouse H. Modes of Religiosity: a cognitive theory of religious transmission. AltaMira Press: Walnut Creek, CA.; 2004.

5. Whitehouse H, Hodder I. Modes of Religiosity at Çatalhöyük. In: Hodder I, editor. Religion in the Emergence of Civilization. Cambridge: Cambridge University Press; 2010. pp. 122–145.

6. Adams ES, Mesterton-Gibbons M. Lanchester’s attrition models and fights among social animals. Behav Ecol. 2003;14: 719–723. doi:10.1093/beheco/arg061

7. Lanchester F. Aircraft in warfare: The dawn of the fourth arm. London: Constable & Co; 1916.

8. Johnson DDP, MacKay NJ. Fight the power: Lanchester’s laws of combat in human evolution. Evol Hum Behav. Elsevier Inc.; 2015;36: 152–163. doi:10.1016/j.evolhumbehav.2014.11.001

9. Dunbar RIM. Neocortex size as a constraint on group size in primates. J Hum Evol. 1992;22: 469–493.

10. Turchin P, Gavrilets S. Evolution of complex hierarchical societies. Soc Hist Evol. 2009;8: 167–198.
