# Supplementary figures and images for: A Life-Cycle Model of Human Social Groups Produces a U-Shaped Distribution in Group Size
Source: PLoS One. 2015 Sep 18;10(9):e0138496. doi: 10.1371/journal.pone.0138496 (PMC4575040; doi:10.1371/journal.pone.0138496)

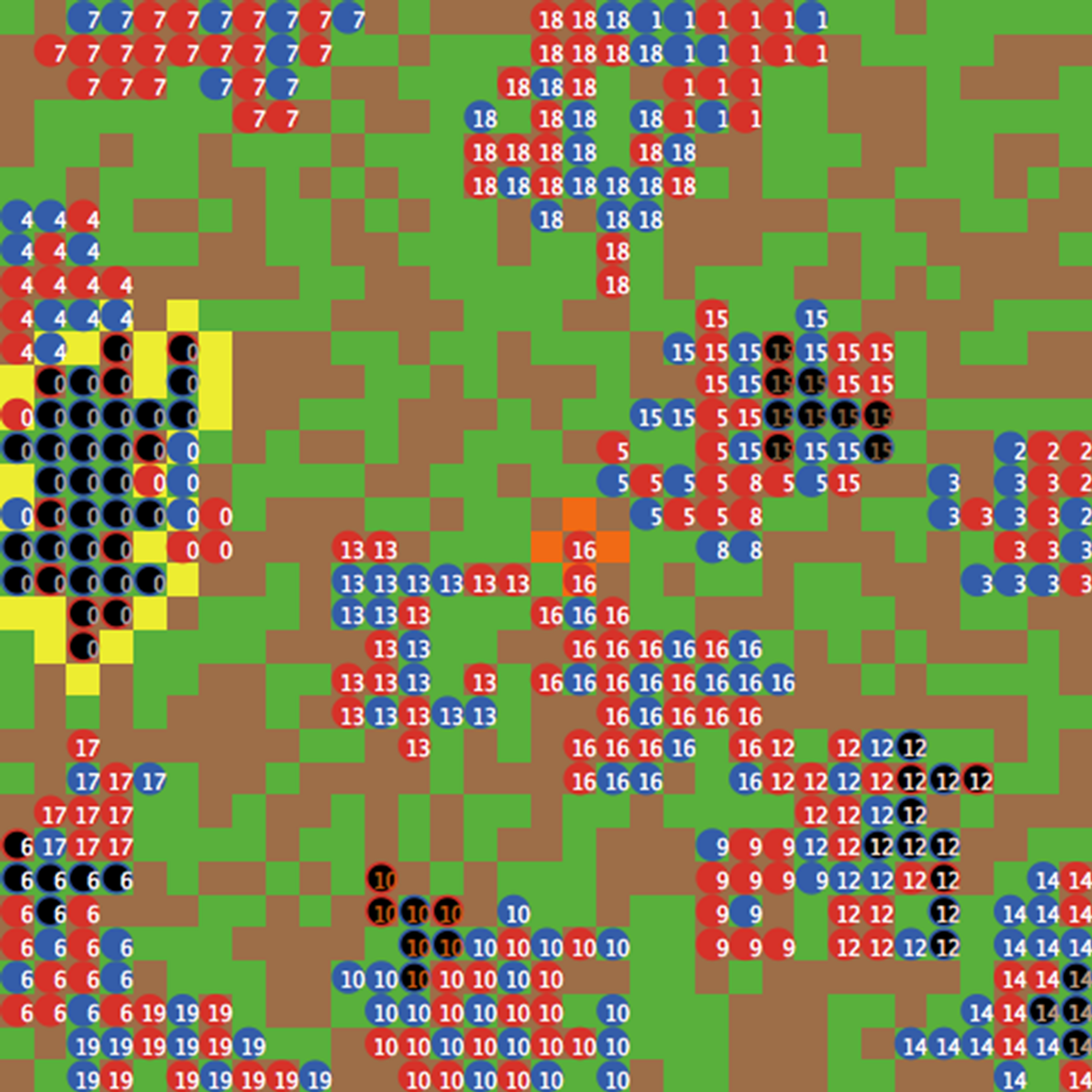

Supplement: S1 Fig — Neighboring patches of a MCG are shown as yellow and that of a SCG as orange. Low-productivity patches are shown as brown squares and high-productivity patches as green squares. SCGs are represented as blue or red circles depending on the productivity of the patch they occupy. The circles with black filling are part of MCGs. Each group has an ID number as a label. Each MCG has a unique label color. (TIF) [file pone.0138496.s002.tif]

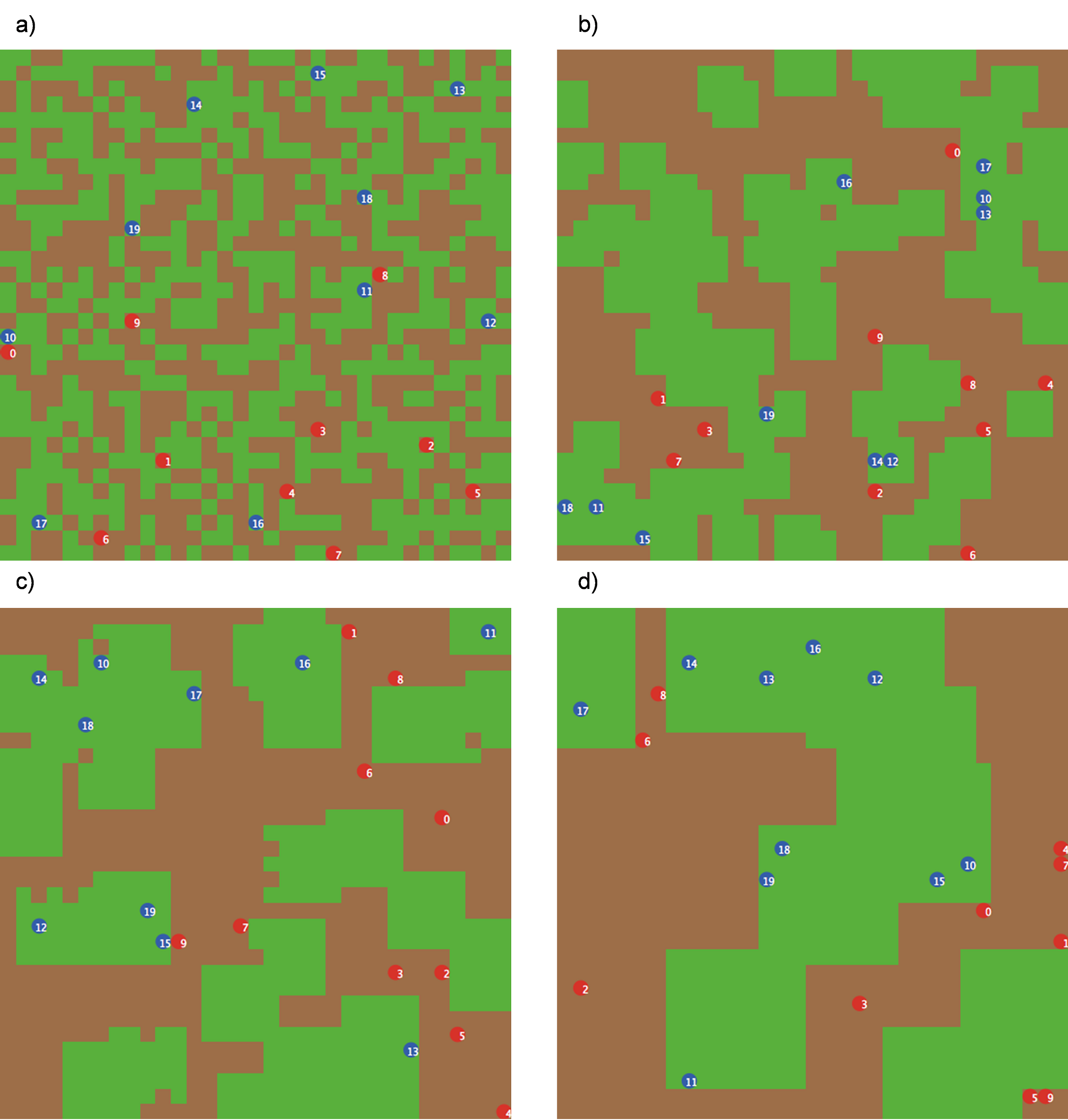

Supplement: S2 Fig — a) HP and LP patches are randomly distributed. b) Aggregation of at least 9 HP patches. c) Aggregation of at least 49 HP patches. d) Aggregation of at least 81 HP patches. (TIF) [file pone.0138496.s003.tif]

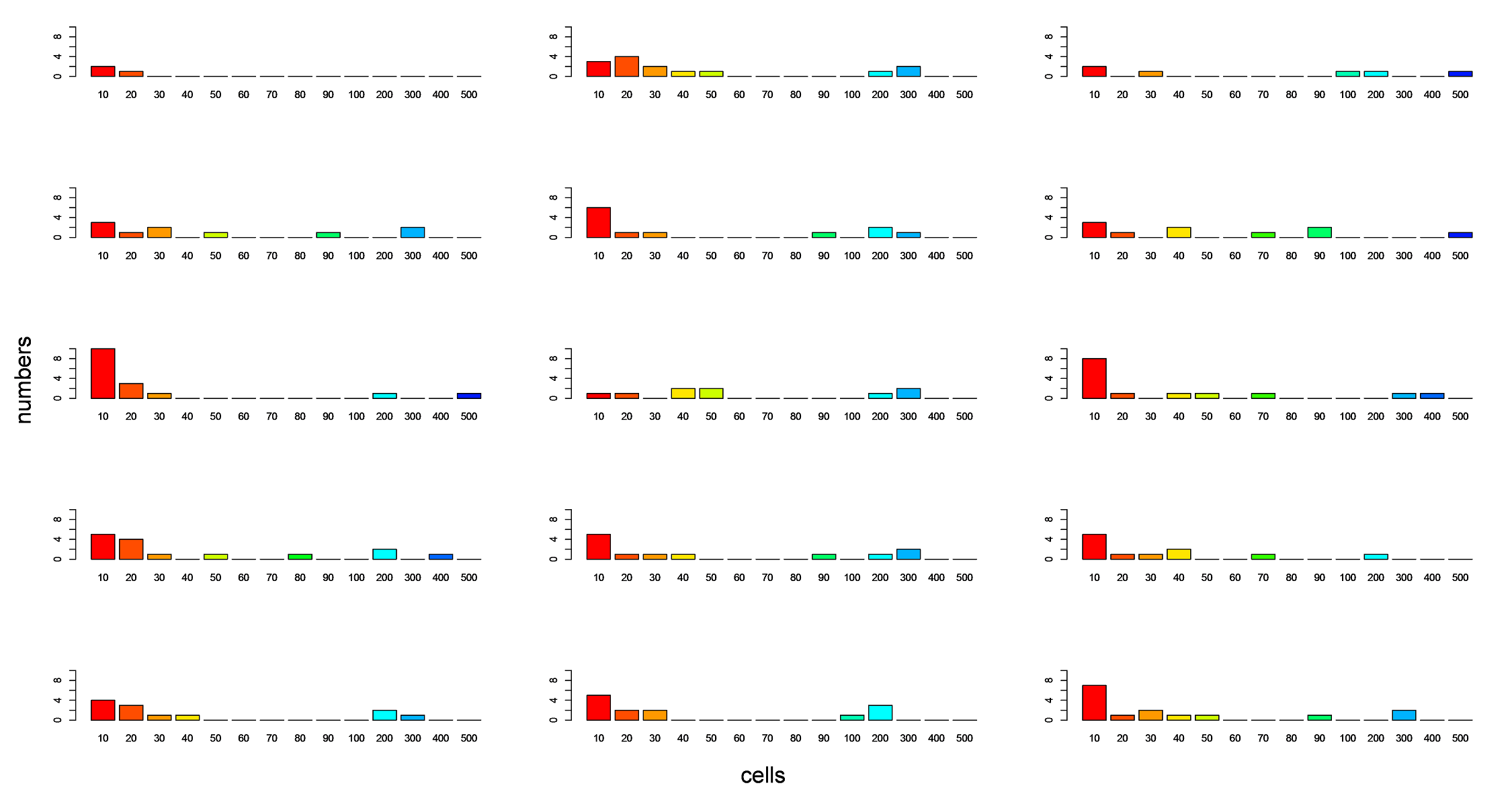

Supplement: S3 Fig — 10 indicates the numbers between 2 to 10, 20: 11 to 20, 30: 21 to 30, …, 200: 101 to 200, 300: 201 to 300 and so on. Note that the replicate shown by the plot on the top left side had a MCG that dominated the landscape with 836 component cells (occupying 77% of the landscape). In general, many MCGs had less than 20 cells and a few others expanded on the landscape with more than 100 cells. (TIF) [file pone.0138496.s004.tif]

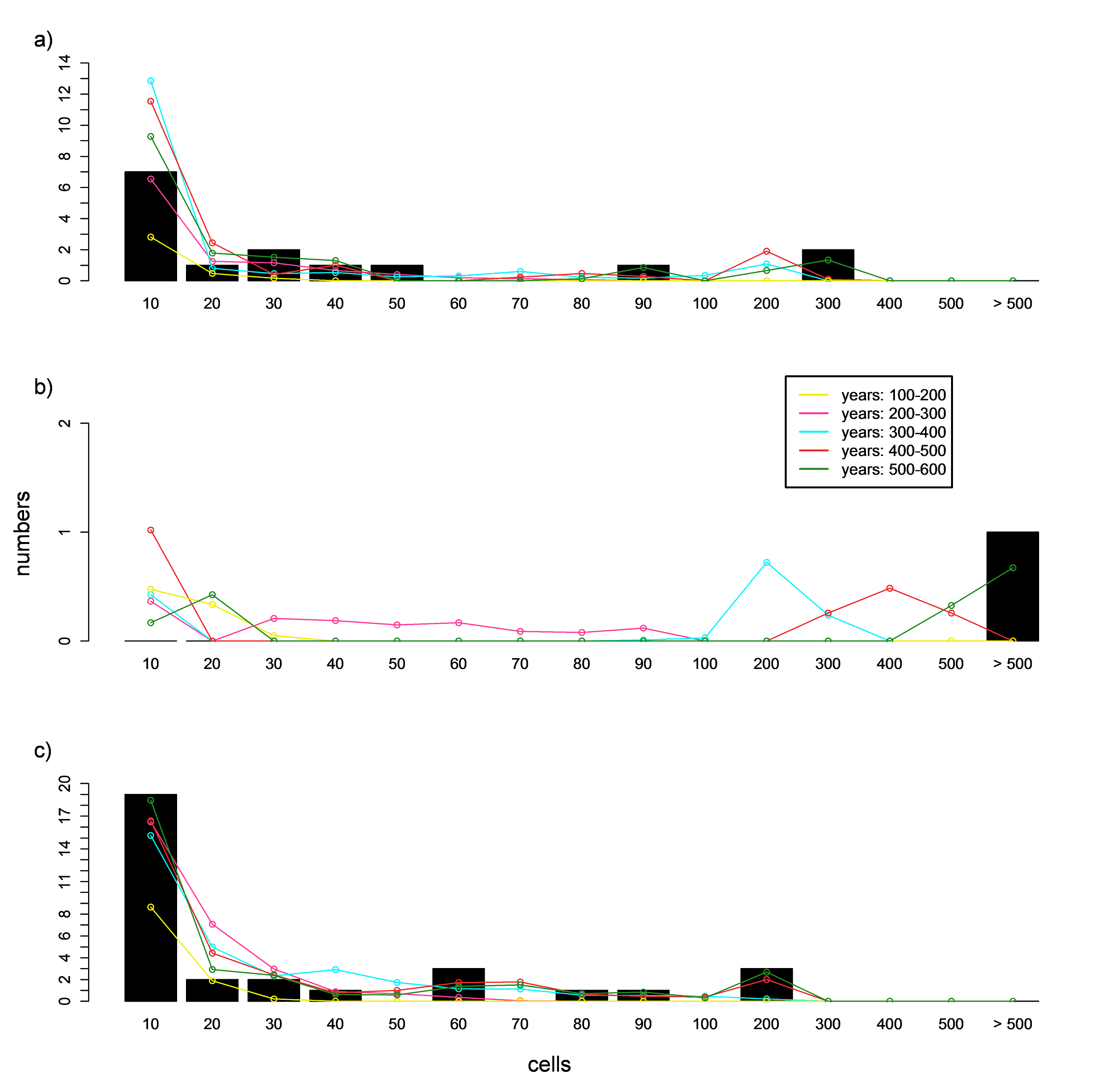

Supplement: S4 Fig — The black bars show the frequencies at t = 600. a) LP/HP patch ratio = 1:1 (default conditions). b) LP/HP patch ratio = 3:1. c) LP/HP patch ratio = 1:3. (TIF) [file pone.0138496.s005.tif]

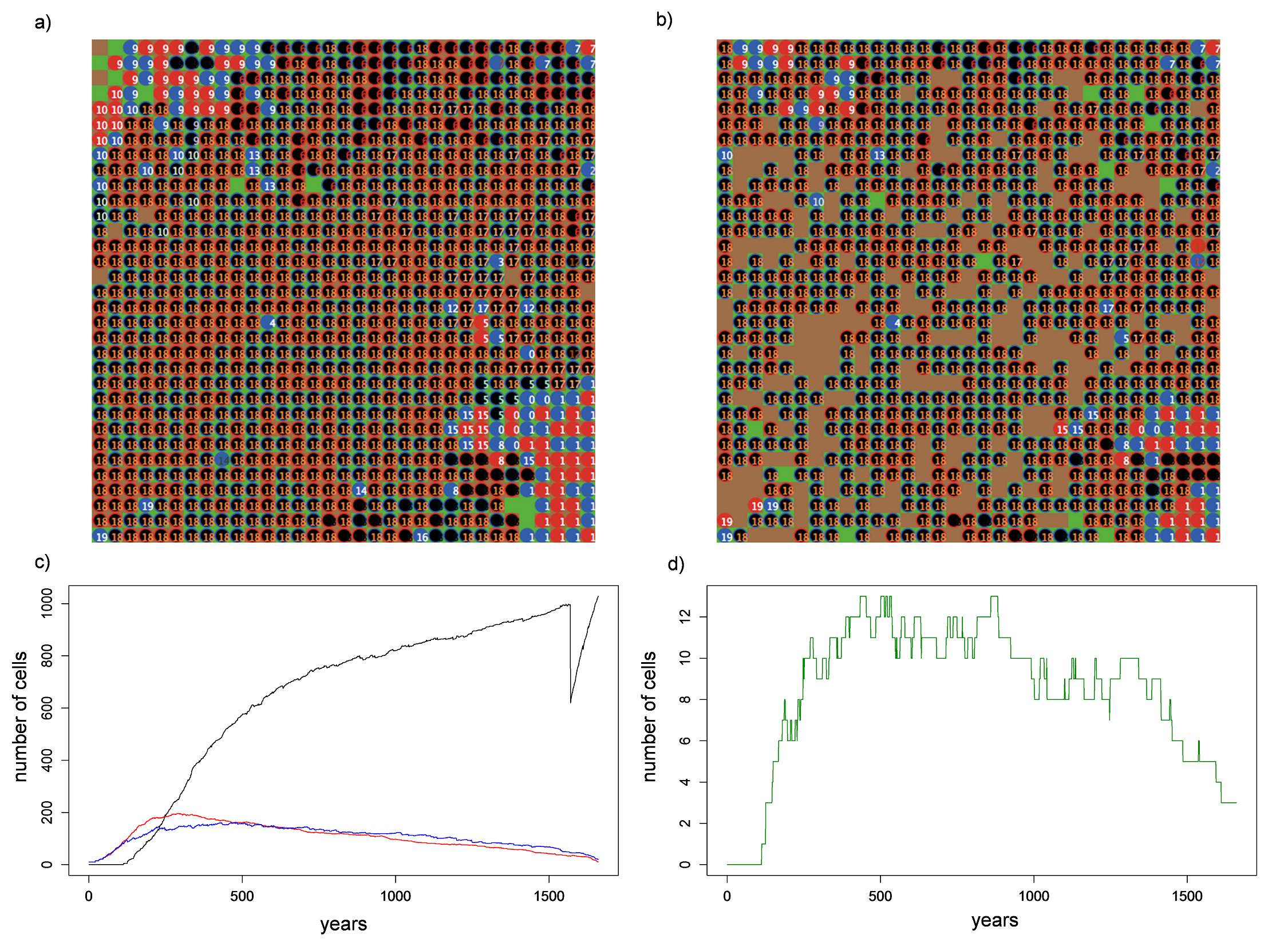

Supplement: S5 Fig — a) A snapshot from NetLogo at t = 1400. The MCG with ID number = 18 (MCG-18) occupies 69% of the landscape with 756 constituent cells. b) A snapshot at t = 1600 shows that MCG-18 is more spread over the landscape, however it has also lost some of its cells (mainly the cells on LP patches) over 200 years. c) Change in the numbers of cells belonging to MCGs (black line), red SCGs (red line) and blue SCGs (blue line) over 1660 years reveals the sudden decrease in the number of cells belonging to MCG-18 at t = 1570 (this is because MCG-18 lost 394 constituent cells due to a war, but rapidly recovered, reaching to the 1012 cells, occupying 93% of the landscape at the end of 1660 years). d) Change in the number of MCGs over the 1660 years of the simulation. (TIF) [file pone.0138496.s006.tif]

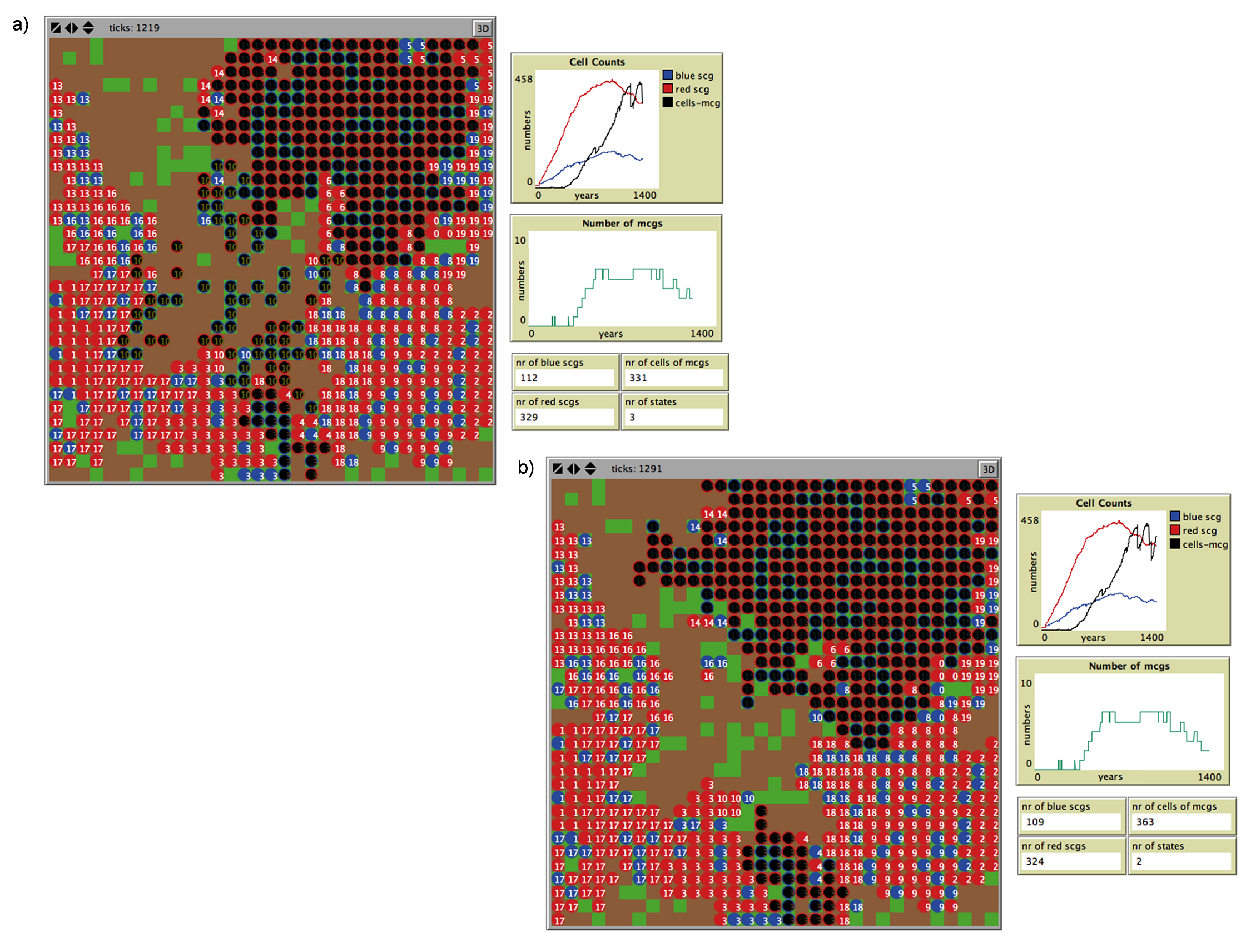

Supplement: S6 Fig — The initial conditions are the same as the default condition, except for the LP/HP patch ratio = 3:1. a) At t = 1219 the MCG with the black ID color (ID number = 15) begins to dominate the landscape. The snapshot is taken just after a war between the MCG with the ID = 15 and the MCG with the ID = 10. Sharp decreases in the black line (cell counts) indicate the loss of the cells belonging to MCGs after two consecutive wars. b) At t = 1291, the MCG with the ID = 10 perishes and MCG with the ID = 15 continues expanding. (TIF) [file pone.0138496.s007.tif]

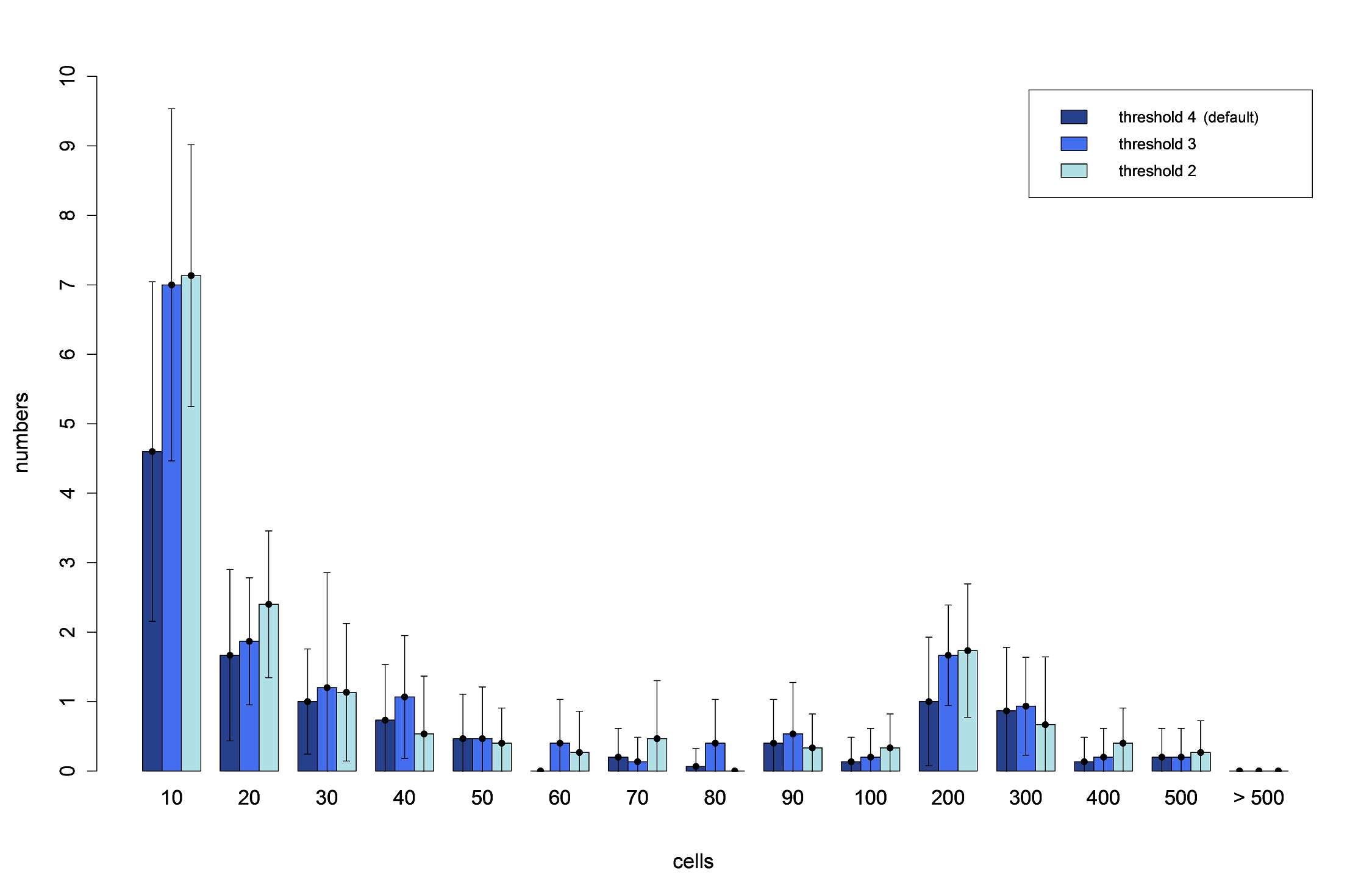

Supplement: S7 Fig — The bars show frequencies of MCGs with respect to the number of their component cells averaged over 15 replicates. Dark blue bars represent frequencies for the experiments where threshold for fusion = 4 (default condition). Bars in the middle represent those with the threshold of 3 and light blue bars those with the threshold of 2. 10 indicates the numbers of cells between 2 to 10, 20: 11 to 20, 30: 21 to 30, …, 200: 101 to 200, 300: 201 to 300, …, > 500: more than 500. Vertical bars mark standard deviations. (TIF) [file pone.0138496.s008.tif]

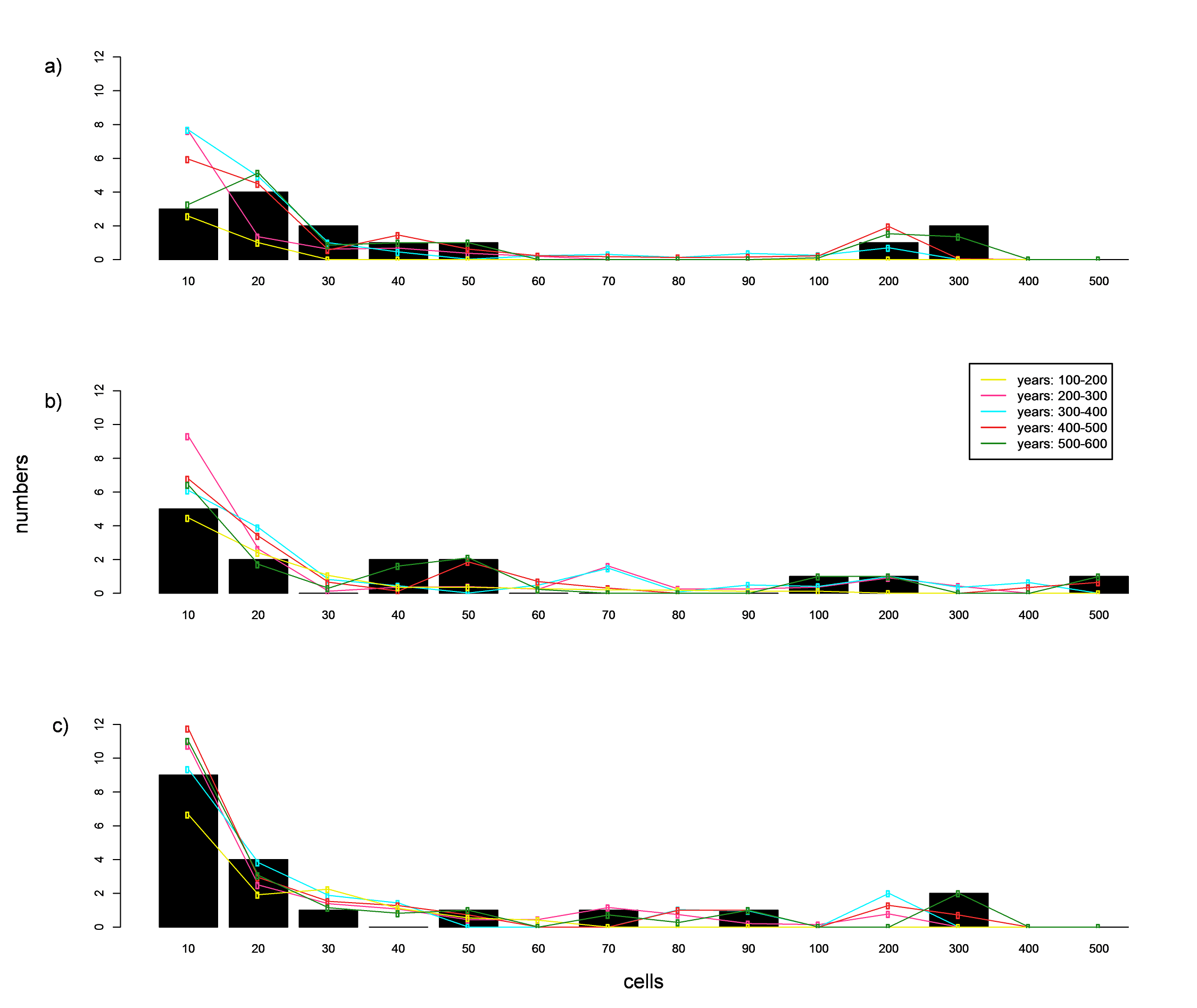

Supplement: S8 Fig — The black bars show the frequencies at t = 600. a) Threshold for fusion = 4 (default condition). b) Threshold for fusion = 3. c) Threshold for fusion = 2. (TIF) [file pone.0138496.s009.tif]

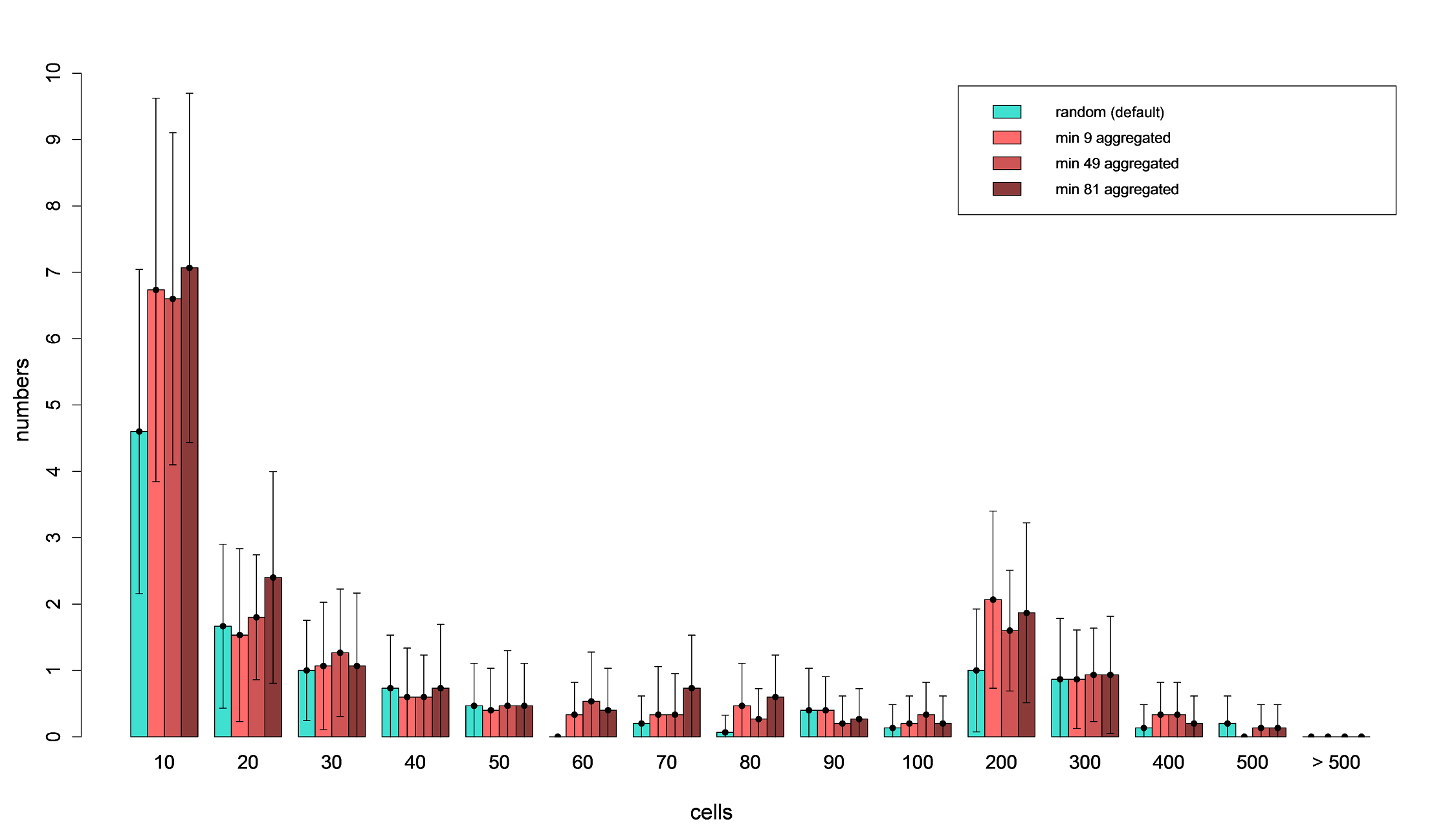

Supplement: S9 Fig — The bars show frequencies of MCGs with respect to the number of their component cells averaged over 15 replicates. The turquoise bar represents mean frequencies for the experiments where LP and HP patches are randomly distributed (default condition). The red bars represent different levels of aggregation; at least 9, 49 and 81 HP patches in clusters from left to right respectively. 10 indicates the numbers of cells between 2 to 10, 20: 11 to 20, 30: 21 to 30, …, 200: 101 to 200, 300: 201 to 300, …, > 500: more than 500. Vertical bars mark standard deviations. (TIF) [file pone.0138496.s010.tif]

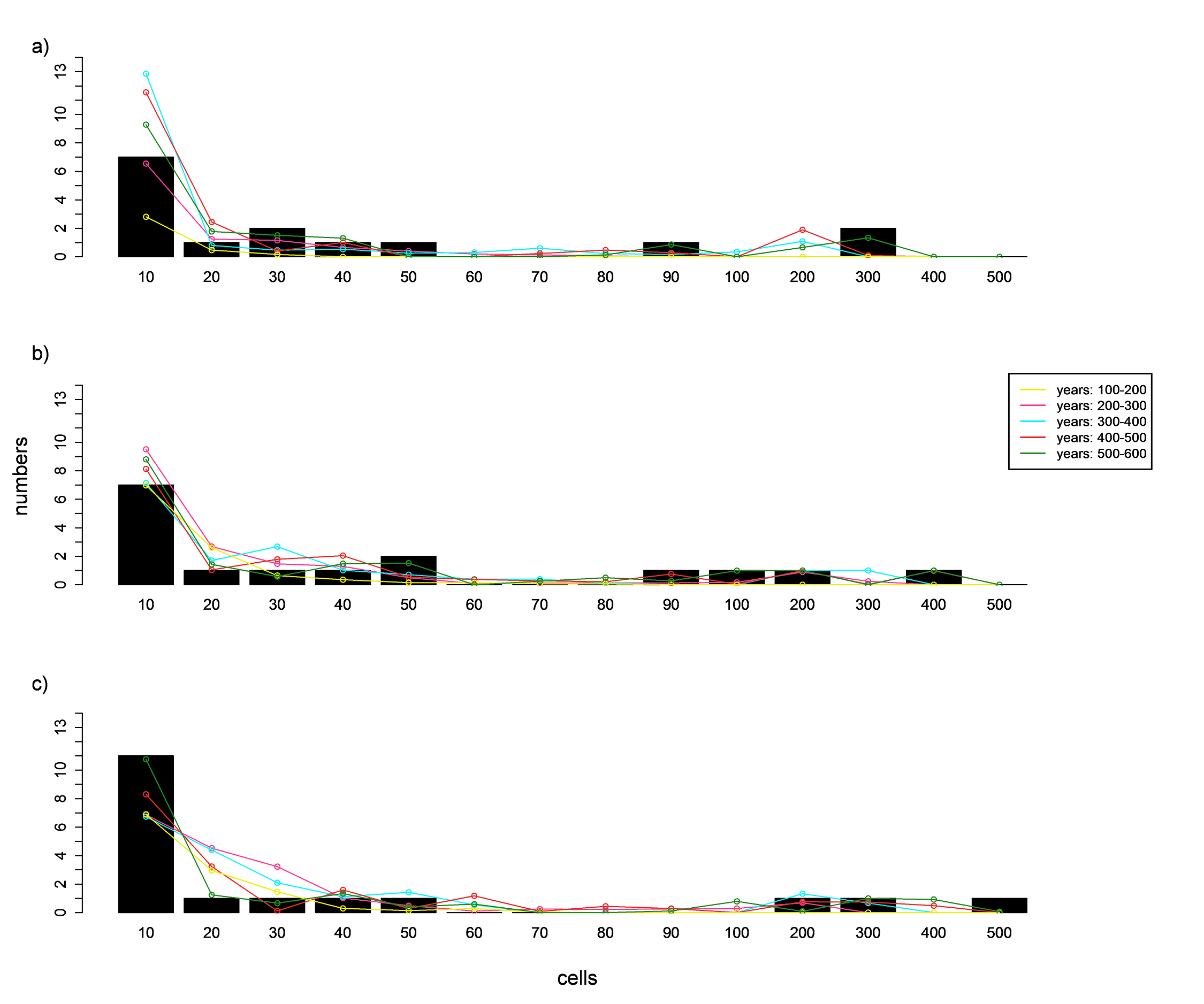

Supplement: S10 Fig — The black bars show the frequencies at t = 600. a) LP and HP patches are randomly distributed (default condition). b) at least 49 HP patches are aggregated. c) at least 81 HP patches are aggregated. Other conditions are the same as the default condition. (TIF) [file pone.0138496.s011.tif]

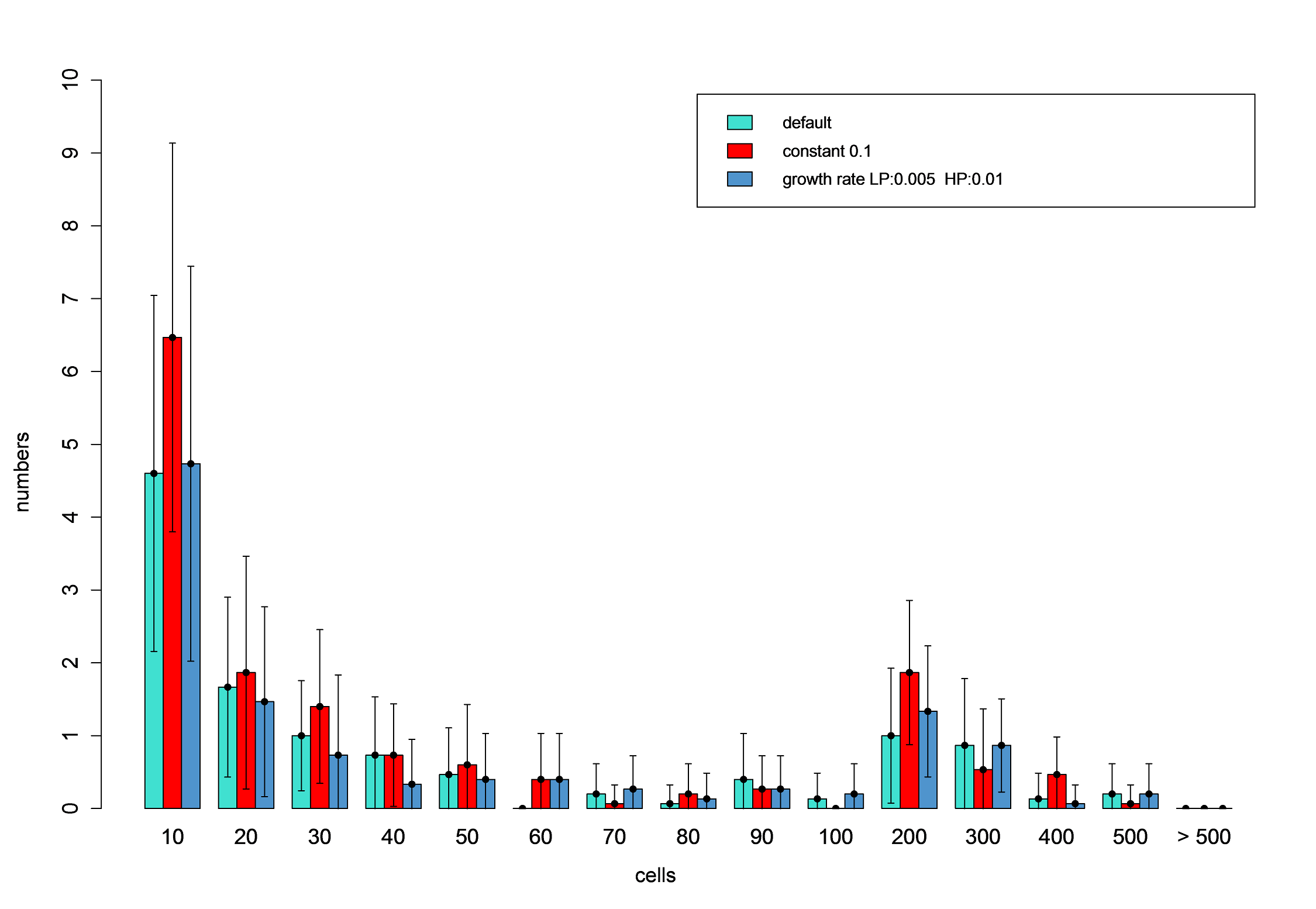

Supplement: S11 Fig — The bars show frequencies of MCGs with respect to the number of their component cells averaged over 15 replicates. Turquoise bars represent mean frequencies for the default condition. Red bars represent the experiments where constant (c) was 0.1. Blue bars represent the condition where HP patch growth rate was set to 0.01 and LP patch growth rate was 0.005. 10 indicates the numbers of cells between 2 to 10, 20: 11 to 20, 30: 21 to 30, …, 200: 101 to 200, 300: 201 to 300, …, > 500: more than 500. Vertical bars mark standard deviations. (TIF) [file pone.0138496.s012.tif]

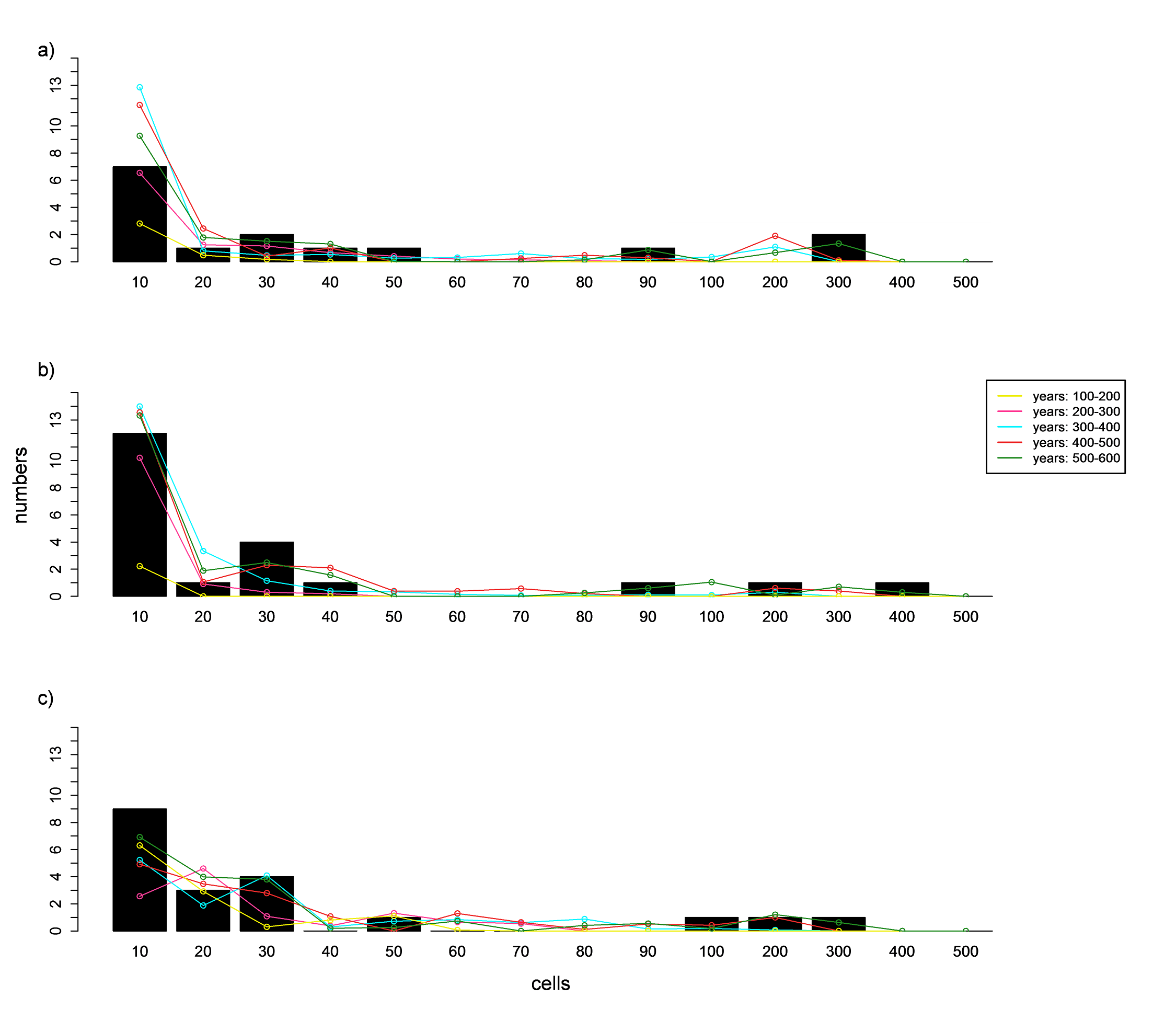

Supplement: S12 Fig — The black bars show the frequencies at t = 600. a) Growth rate on LP patches = 0.01, on HP patches = 0.03, constant (c) = 0.001 (default condition). b) Growth rate on LP patches = 0.01, on HP patches = 0.03, constant (c) = 0.1. c) Growth rate on LP patches = 0.005, growth rate on HP patches = 0.01, constant (c) = 0.001. All other initial conditions are the same as the default conditions. (TIF) [file pone.0138496.s013.tif]
